# 3D Extrusion Printing of Biphasic Anthropomorphic Brain Phantoms Mimicking MR Relaxation Times Based on Alginate-Agarose-Carrageenan Blends

**DOI:** 10.1021/acsami.2c12872

**Published:** 2022-10-21

**Authors:** David Kilian, Wolfgang Kilian, Adriano Troia, Thanh-Duc Nguyen, Bernd Ittermann, Luca Zilberti, Michael Gelinsky

**Affiliations:** † Centre for Translational Bone, Joint and Soft Tissue Research, Faculty of Medicine Carl Gustav Carus, Technische Universität Dresden (TUD), Dresden01307, Germany; ‡ Physikalisch-Technische Bundesanstalt (PTB), Berlin10587, Germany; § Istituto Nazionale di Ricerca Metrologica (INRiM), Turin10135, Italy

**Keywords:** additive manufacturing, gray matter, white matter, magnetic resonance imaging, hydrogels, relaxation times, 3D plotting, bioprinting

## Abstract

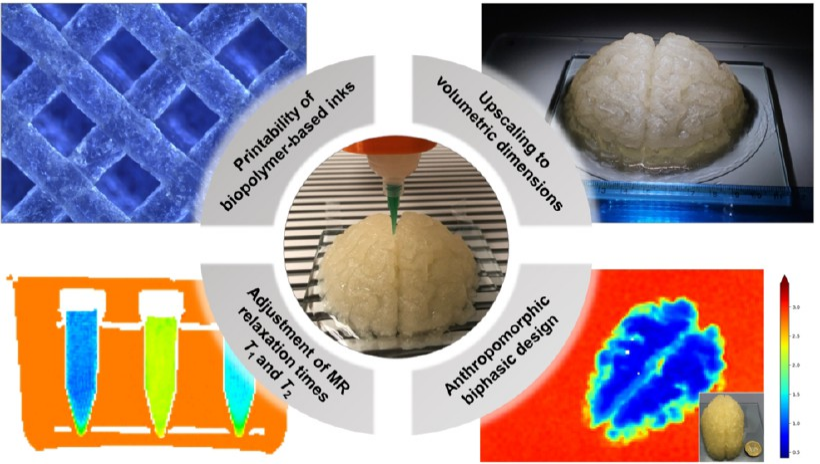

The availability of adapted phantoms mimicking different body parts is fundamental to establishing the stability and reliability of magnetic resonance imaging (MRI) methods. The primary purpose of such phantoms is the mimicking of physiologically relevant, contrast-creating relaxation times *T*_1_ and *T*_2_. For the head, frequently examined by MRI, an anthropomorphic design of brain phantoms would imply the discrimination of gray matter and white matter (WM) within defined, spatially distributed compartments. Multichannel extrusion printing allows the layer-by-layer fabrication of multiple pastelike materials in a spatially defined manner with a predefined shape. In this study, the advantages of this method are used to fabricate biphasic brain phantoms mimicking MR relaxation times and anthropomorphic geometry. The printable ink was based on purely naturally derived polymers: alginate as a calcium-cross-linkable gelling agent, agarose, ι-carrageenan, and GdCl_3_ in different concentrations (0–280 μmol kg^–1^) as the paramagnetic component. The suggested inks (e.g., 3Alg-1Agar-6Car) fulfilled the requirements of viscoelastic behavior and printability of large constructs (>150 mL). The microstructure and distribution of GdCl_3_ were assessed by scanning electron microscopy (SEM) with energy-dispersive X-ray spectroscopy (EDX). In closely monitored steps of technological development and characterization, from monophasic and biphasic samples as printable inks and cross-linked gels, we describe the construction of large-scale phantom models whose relaxation times were characterized and checked for stability over time.

## 1 Introduction

Magnetic resonance imaging (MRI) has a tremendous value and impact in today’s healthcare. It allows precise diagnosis without harmful radiation exposure,^1^ purely based on the contrast arising from signal relaxation differences in various tissue types weighted during the image acquisition. With high resolution achievable through well-established magnetic field strengths, it is a safe noninvasive diagnostic tool. Increasing accessibility and ongoing technical developments allow for the detection of morphological and structural changes in many different pathological situations. The method is used for diagnosis of soft tissue pathologies,^2^ tumor detection,^3^ or for the design of patient-individualized implants and tissue substitutes for tissue interfaces.^4^ Typically, identification of tissue alterations still relies on the visual perception of the radiologist or physician observing the imaging data via visualizing software solutions. However, quantification of the tissue parameters that underlie the MR image generation (qMRI) in the future might allow a fully automated, more sensitive, and less biased evaluation of the *in vivo* situation.^5,6^ This has been proven for the detection of adverse effects on the myocardium of breast cancer patients after radiation therapy^6^ or for differentiation of tumor malignancy by electrical conductivity.^7^ Additionally, increasingly new imaging strategies like MR fingerprinting or machine learned image reconstruction are established with the aim of fast and reliable qMRI.^8^ All these new strategies require validation and calibration of devices and algorithms. For such validation steps, stable, reliable phantoms resembling not only tissue-specific nuclear magnetic resonance (NMR) properties (relaxation times) but also anatomical geometries are required. In this context, metrological research focuses on the development of novel MRI phantoms^9,10^ for use by scanner manufacturers, research institutions, public validation agencies, hospitals, and other relevant sites. An often-cited example for a valuable phantom aims for internally reflecting a human head structured into gray matter (GM) and white matter (WM) of the brain with a filled-up space to the surrounding cranial bone with the cerebrospinal fluid (CSF);^11^ the soft matter compartment consists of cell bodies (GM) and myelinated axons (WM) of neurons.

Conventional MRI phantoms primarily consist of gel-based solutions admixed with paramagnetic ions such as NiCl_2_, MnCl_2_, or GdCl_3_.^12^ Typically one type of ion has been chosen for adjustments, while, in later studies, two of these paramagnetic ions have been applied simultaneously to trigger both MR relaxation times *T*_1_ and *T*_2_ in parallel.^13^*T*_1_ defines the longitudinal relaxation time, the return time of the excited proton spin magnetization to its thermal equilibrium; *T*_2_ is the transverse relaxation time indicating the time it takes for the coherent spin precession to dephase due to the spin–spin interaction.^14,15^ The aim of MRI phantoms is to resemble these relaxation times found *in vivo* by an artificial construct. Standard commercial MRI reference phantoms consist of individually sealed vials with paramagnetic solutions, forming highly stable, very valuable standard systems, such as based on a nine- (T1MES)^16^ or even a 57-element fiducial array.^17^ However, for the advancement of quantitative methods with a high spatial resolution in anatomic geometries, novel anthropomorphic phantoms will be indispensable. So far, such structures have required the usage of casting molds with walls in between different compartments representing the anthropomorphic shape^18^ as approaches without separating layers lacked stability of the spin-relaxation times.^19^ For fast, easy hardening and long-term stability, the materials filled into such objects are mainly based on biopolymer hydrogel networks with different gelling mechanism. Thermal gelation plays a dominant role as it allows a rapid and homogeneous formation of cross-links inside the casted structures, such as for agar, agarose or gelatin.^20−22^ However, none of these has been able to combine an ideal adjustment of *T*_1_ and *T*_2_ with an adequate, stable contrast at the interface of GM and WM without additional barriers.

Some earlier approaches used polysaccharide blends based on agarose, for instance, in combination with paramagnetic ions to effectively adjust the relaxation parameters of phantoms in particular ranges.^21^ The effects rely on an altered network density with varying water contents and the effect of gadolinium, nickel, or manganese as paramagnetic ions. In this way, Yoshimura et al. already showed that with a higher overall concentration of *T*_1_ modifier GdCl_3_ (in μmol kg^–1^), reduced polymer concentrations of *T*_2_ modifier (here: agarose) were needed to efficiently reduce *T*_2_. Similar results were confirmed for agarose as the *T*_2_-modifier and NiCl_2_ as the corresponding *T*_1_-modifier.^16^ These components, agarose (as the *T*_2_-relaxation modifier) and GdCl_3_, can also be combined with carrageenan as an (additional) gelling agent.^23^ Gadolinium chelated to a carrier ligand has been widely used as a rather biocompatible MR contrast agent in low concentrations.^24^ Altermatt and co-workers introduced an alternative approach for anthropomorphic shapes in biphasic phantoms with a rather stable GM/WM interface by distinct MnCl_2_ contents; however, this was possible only by using a separating wax layer at the compartment interface.^19^ Some approaches also manage to approach the physiological values for conductivity and/or permittivity,^23^ which was not the main focus of the present study.

Techniques of additive manufacturing (AM) still play a minor role, as AM is merely used for the fabrication of casting molds: Yunker and co-workers have evaluated the relevance and suitability of materials for the 3D printing of MRI phantoms,^25^ while Gopalan et al. 3D printed a cast that can be manually filled up with a low viscosity hydrogel for biphasic structures.^18,26^ Another study suggested a 3D-printed, PLA-based shell for filling up the phantom structure in order to simulate transcranial magnetic stimulation.^27^ Other popular alternative materials are silicones,^28^ while other approaches describe the casting or printing of hydrogel phases consisting of polysaccharides such as gelatin to match the mechanical properties of brain tissue,^29,30^ or their combination with polymers such as poly vinyl alcohol (PVA),^30^ or different types of silicone.^25,31−33^

Over previous years, extrusion-based AM of hydrogel systems^34^ has gained large interest for various other applications. One of the most prominent ones, of whose rapid advancements other branches of AM can benefit from, is tissue engineering.^35^ Here, AM allows the patient- and defect-specific design and fabrication of scaffolds and tissue substitutes. Hydrogel printing is further applied toward electronic devices or magnetic actuator constructs.^36^ The selected materials can be synthetic, but are rather often based on biopolymer blends, similar to the generation of conventional phantom models for MRI. Unlike casting methods, AM allows the combination of material phases with a spatially defined distribution by multichannel printing, without the need for either a casting mold or rigid separation walls. However, aside from the AM-compatible properties of the pastes that need to be ensured, the effect on the diffusive behavior of material components and paramagnetic ions at a possible GM/WM interface needs to be investigated.

Strategies, in this context, can be adapted from bioprinting concepts for large construct fabrication in clinically relevant dimensions.^37,38^ However, in our case, cytocompatible cross-linking mechanisms, which are a crucial factor and often a significant limitation in biofabrication,^39^ can be neglected since no cells are involved in the fabrication process of MRI phantoms. As a consequence, the polymer content can be adjusted with a primary focus on printability and on the desired MR-active properties. Such AM concepts can make it easier to combine two or more materials with a spatially defined distribution in phantoms and can allow the formation of cavities even, which is made possible through the consideration of sacrificial materials.

Therefore, the aim of this study was the development of a novel MRI phantom of multizonal anthropomorphic dimensions by 3D hydrogel printing. In this context, we selected suitable biopolymers to blend to an ink applicable for 3D extrusion printing of stable MRI phantom brain models in clinically relevant dimensions with adjustable MR-active properties (relaxation times *T*_1_ and *T*_2_). As a basis, ionically cross-linkable sodium alginate was chosen which has been typically applied for various hydrogel printing studies^34^ and has also been described in a combination with gadolinium for MRI tracing of drug delivery.^40^ To make this material printable for AM generation of phantoms, it was blended with agarose as a pregelled, stabilizing component of the ink, which can be used to adjust *T*_2_ along with the use of the *T*_1_-modifier GdCl_3_ as demonstrated by Yoshimura and colleagues.^21^ In order to enhance shear thinning properties, a naturally derived thickener (ι-carrageenan) was added. As an additional, natural shear thinning component, ι-carrageenan was chosen to adjust viscoelastic properties for an adequate extrusion of alginate to a stable, cross-linkable strand after deposition,^41^ which is not achievable in its native form.^42,43^

In order to be successful with such a novel, fully 3D-printed multizonal MRI phantom with a resolution of a real brain MRI (typically 1 mm), certain steps and subgoals were defined that will be investigated and covered in this study:

- Adjusting the material properties of a novel alginate-agarose-carrageenan blend to ensure a continuous extrusion with shear thinning properties
- Ensuring the stability of extruded strands and the shape fidelity of printed structures postfabrication
- Establishing a compatible and sustained cross-linking scheme for a stable hydrogel network
- Investigating relaxation times *T*_1_ and *T*_2_ in printable inks and in the corresponding cross-linked hydrogels
- Controlling relaxation times by this novel ink composition and GdCl_3_ supplementation
- Considering anthropomorphic 3D design for generation of the first 3D-printed hydrogel phantom with such defined geometries
- Implementing a biphasic (anthropomorphic) GM-WM phantom design into the printing process
- Generating a stable, MR-distinguishable GM-WM interface with zonally defined relaxation times in GM and WM, for the first time without an additional GM-WM barrier
- Upscaling of the phantom fabrication process toward a phantom volume ≥120 mL

## 2 Materials and Methods

### 2.1 Material Sources and Ink Preparation

All the ink compositions were based on the same three biopolymer components: sodium alginate was purchased in the form of alginic acid sodium salt from brown algae (Sigma-Aldrich, Germany, #71238). This alginate has an M:G ratio of 1:2 (homopolymeric copolymer content; M = β-d-mannuronate, G = α-l-guluronate). UltraPure agarose was purchased from ThermoFisher Scientific, USA; #16500). Highly purified iota (ι)-carrageenan was obtained by two different sources: TCI Chemicals, Germany (#C1805) and Fluka/Sigma-Aldrich (#22045, approximately 14 mPa s).

The respective weight percent (wt %) of alginate was dissolved in double-deionized water. At approximately 100 °C, agarose powder was added and dissolved by magnetic stirring at approximately 400 rpm in a closed jar. After cooling of the blend, on the day before sample fabrication, the respective weight percents of ι-carrageenan were added and stirred in manually. Gadolinium(III)chloride (GdCl_3_, anhydrous powder, Sigma-Aldrich, USA, #439770) was applied as the main agent for modifying relaxation times *T*_1_ and *T*_2_. GdCl_3_ was dissolved at respective concentrations to obtain a 100× stock solution that was added to the inks as 10 μL per 1.0 g of ink. To avoid spoilage and associated potential degeneration during long-term storage, we added 0.1 wt % *ProClin150* (Sigma-Aldrich, USA) as a fungicide where required. After that, this ink was used to prepare samples as described in the following sections. If not stated otherwise, molded or 3D-printed samples were cross-linked by addition of excess amounts of 1 M CaCl_2_ solution for 20 min (by pipetting the cross-linking solution on top of the printed construct). Table 1 summarizes the terminology for applied ink compositions based on different concentrations (*x*/*y*/*z* wt %) of alginate (Alg), agarose (Agar), and carrageenan (Car), with possible concentrations of GdCl_3_ (in μmol kg^–1^) added in brackets behind the biopolymer composition. Throughout the article, we will be referring to the pastelike, printable materials as “inks”, and to the cross-linked inks and cross-linked constructs (after CaCl_2_ treatment) as “gels” or “hydrogels”.

**Table 1:** Primary Ink Compositions *x*Alg**-***y*Agar-*z*Car Listed with Their Terminology, Concentrations, and Respective Component Functionalities Inside the Ink

	alginate (wt %)	agarose (wt %)	ι-carrageenan (wt %)	GdCl_3_ (μmol kg^–1^)	fungicide
function	calcium-cross-linkable network	pregelled component	thickener for shear thinning	*T*_1_ modifier	prevent contamination
3Alg-1Agar	3	1			(±0.1% *ProClin150*)
3Alg-1Agar-6Car	3	1	6		
3Alg-1Agar-9Car	3	1	9		
3Alg-0.5Agar	3	0.5			
3Alg-0.5Agar	3	0.5			
3Alg-0.8Agar-3Car	3	0.8	3		
3Alg-0.8Agar-6Car	3	0.8	6		
3Alg-1Agar-6Car (c_Gd_)	3	1	6	*c*_Gd_ = 0/70/140/210/280	
3Alg-0.5Agar-3Car	3	0.5	3		
3Alg-0.5Agar-6Car	3	0.5	6	210	

	alginate (%)			methylcellulose (%)	
3Alg-9MC	3			9	

### 2.2 Assessing Viscoelastic and Rheological Properties of Developed Inks

Shear-thinning behavior and a viscosity high enough for stable strand extrusion are essential parameters for extrusion-based 3D printing. A plate-plate rheometer setup (diameter 35 mm; Rheotest, Medingen, Germany) with a distance of 0.1 mm was used for all rheological analyses. Shear ramp analysis was conducted for all relevant ink compositions with a continuous shear increase of 0–100 s^–1^ within 600 s. The shear recovery mechanism was investigated for the most relevant ink compositions by an assigned iterative protocol of alternating changes of high and low shear rate: 5 s^–1^ for 120 s, 100 or 500 s^–1^ for 60 s, 5 s^–1^ for 180 s, 100 s^–1^ or 500 s^–1^ for 60 s, and 5 s^–1^ for 180 s. Shear stress and viscosity were monitored throughout these intervals.

### 2.3 3D Extrusion Printing: Assessing Ink Printability and Strand Swelling Behavior

The inks were filled into cartridges using a spatula. Prior to printing, 10 or 30 mL cartridges were centrifuged for 20 min at 2000–3000 rpm to remove air bubbles from the inks. BioScaffolder 3.1 (GeSiM, Radeberg, Germany) was used for monophasic or multiphasic construct printing (by multichannel extrusion) for volumes up to 30 mL per phantom when using only one printing head and one cartridge. The ink was pneumatically extruded from the cartridge through conically shaped nozzles with an inner outlet diameter of 840 μm. With this diameter, speed (time to complete one large phantom) and resolution for volumetric dimensions were both considered in an acceptable manner. For evaluating the extrusion and phantom fabrication behavior, structures of a square-shaped geometry (14 × 14 mm^2^ × 4 layers of perpendicular strands) were manufactured, with an adjusted mean infill strand distance of 2.3 mm.

Images of these extruded square-shaped, open porous samples were taken using a Leica M205C stereo microscope equipped with a Leica DFC295 camera (Leica, Germany). Strand width ratio was calculated as the ratio of resulting strand diameter prior to cross-linking and the inner nozzle diameter (840 μm). At different time points (on day 0 before and after cross-linking, day 2, day 4, and day 8), images were taken for analyzing swelling behavior in open-porous structures, during storage in different solutions for 8 days: (a) H_2_O, (b) 1 M CaCl_2_, (c) 100 mM CaCl_2_, and (d) 10 mM CaCl_2_. During storage in the listed solutions, ideal conditions for long-term storage were examined. With microscopy images, strand diameters at randomly chosen positions in three replicates (total *n* ≥ 15) were measured and evaluated using *ImageJ* software.

### 2.4 Sample Preparation for MR Measurements

For measurements of MR relaxation times in small samples, non-cross-linked inks were filled into 5 mL Eppendorf tubes. The samples for measurements after cross-linking were 3D printed (see above) with a 100% infill pattern in the shape of the cylindrical Eppendorf tube (diameter *d* = 14 mm, height *z* = 30 mm, 35 layers), cross-linked with 1 M CaCl_2_ and placed inside the Eppendorf tube with 100 mM cross-linking solution as surrounding medium to avoid air inclusions. For MR measurements, all Eppendorf tubes were put into a rack which was placed in a 2.7 L food storage box filled with a low concentrated hydroxyethyl cellulose solution (HEC; 1 wt% in 1.33 g L^–1^ NaCl and 0.66 g L^–1^ CuSO_4_) for coil loading.

For anthropomorphic monophasic and biphasic samples, a section of a brain slab with a thickness of 11 mm and a maximum length and width of 57 mm and 39 mm was selected from a CAD file derived from a segmented, anonymous MRI data set, combined and visualized using Microsoft 3D Builder. For monophasic printing, GM and WM volumes were fused to one common architecture prior to slicing of the model for printing. Samples were stored in sealable 75 g salve containers for sample shipment. For MR characterization, the monophasic samples were all fixed in an Eppendorf rack. This rack was placed in the 2.7 L storage box, which was filled with a 0.11 M CaCl_2_ solution this time. The five biphasic samples were placed in individual 30 mL jars with dimensions just slightly larger (diameter 51 mm; height 14 mm) than the sample size; those were filled with a solution of 0.1 wt% ProClin-150 and 0.1 M CaCl_2_ in deionized H_2_O. All these jars were then evenly spaced by plastic rings within a 400 mL plastic jar filled up with the HEC solution as described above. In order to test the impact of a fungicide additive for long-term storage, 0.1% *ProClin150* was added to the MR-active inks prior to sample fabrication.

### 2.5 Scanning Electron Microscopy (SEM) and Energy-Dispersive X-ray Spectroscopy (EDX)

For preparation, samples were dehydrated in a mild vacuum pressure chamber to prevent samples from deformation. A SEM FEI Inspect F FEG (FEI, USA; operated in SEM mode) at a voltage range of 10–30 kV (spot size 3.5, working distance 12.5 ± 0.5 mm) with a field-emission gun was used for microstructural analysis and imaging. This instrument was located at the Istituto Nazionale di Ricerca Metrologica (INRiM), Turin, Italy. In addition, energy-dispersive electron spectroscopy (EDX) mapping was performed at a voltage of 30 kV. EDX signals were used to analyze and visualize/map the structure and distribution of elements throughout the ink and, in particular, of Gd and Cl as ions of the *T*_1_-modifying, paramagnetic salt component. The EDX aperture was of 30 μm, while the count/dead time was about 35–40% for all the maps acquired. This described sample preparation was sufficient to prevent charging effects for the suitable time needed for SEM imaging and microanalysis.

### 2.6 Measuring MR Relaxation Times

All MR measurements were performed using a clinical 3 T MR scanner (Siemens Verio, Germany) located at Physikalisch-Technische Bundesanstalt (PTB), Berlin. For determining the two MR relaxation parameters *T*_1_ and *T*_2_, we used the commonly accepted gold-standard MR slice-selective single-echo imaging sequences “inversion–recovery spin–echo” (IR-SE) and “spin echo” (SE), respectively, and the raw complex data were stored for offline analysis.

The MR sequence parameters were adapted to the corresponding phantom size in general comprising slice thicknesses in the range of 4–7 mm and quadratic voxel size with 0.7–0.9 mm lateral length. The repetition time (TR) was always set to 10 s, which was expected to be long enough for all relevant *T*_1_ and *T*_2_ times for full magnetization recovery before the next excitation.

For *T*_1_ time determination with the IR-SE sequence, the inversion recovery (IR) time was varied with values set by 2^*n*^ × 25 ms with n = 0–8 resulting in the range of 25 to 6400 ms in total. For *T*_2_ time measurements in the beginning, a somehow coarse variation in echo-times (TE) was used with values of 15–800 ms. As clear double exponential *T*_2_ relaxation behavior has been observed in other studies,^31^ we have also used a denser sampling of short echo times with TE = 12, 13, 15, 20, 25, 30, 40, 80, 160, 320, and 640 ms for most of the measurements. To exclude the potential effects of drifts over the relatively long overnight measurement, we did not linearly sample the time image acquisition with different TI, as well as TE, times, but used a shuffled mode so that a short value is followed by a long one.

Analysis was done offline using an in-house software capable of reading the raw Siemens data. Prior to the fast Fourier transformation (FFT), which converts the measured *k*-space data into the conventional image space, we applied a 2D symmetric filter centered at the origin of the *k*-space. This filtering is necessary for all MR acquisition applied on samples having an artificial sharp signal variation within the object, which is normally the case for phantoms of different compartments being separated by ridged walls to suppress the well-known Gibbs artifacts.^44^ Even though there are highly sophisticated techniques described to suppress those artifacts,^44^ effective filtering is achieved when using a normalized exponential filter function.
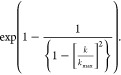
1This function asymptotically reaches zero at the maximum sampled *k*-space values *k*_max_. However, it suppresses signal intensities to a lesser extent than the commonly applied Lorentzian or Gaussian function-based filters. With this exponential filter function, an effective nearest neighbor averaging with a full width of half-maximum of 1.8 pixels is introduced, comparable to a sinc filter function. However, signal smearing decreases more rapidly for distances being more than five pixels away. Thus, an in-plane resolution in the range of 1–2 mm is given in all presented MR images, much smaller than the slice thickness being used (4–7 mm).

After filtering and performing the FFT, a pixel-wise least-squares minimization is applied to the signal variation in dependence of the TI or TE time. The most adequate formula being used to analyze *T*_1_ from complex data is
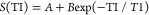
2with *A* and *B* being complex and *T*_1_ as the real fit parameter.^45^ For the analysis of the signal dependence of the SE data with varying TE, we calculated magnitude images and applied the following fit function:
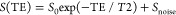
3which is not fully correct to describe the Rice distribution intensity of the noise (*S*_noise_) when calculating the magnitude signal but is considered much more reliable than simply using the exponential decay without the offset. When a clear systematic behavior is seen in the residual, a second exponential decay is added to the function describing the *T*_2_ signal decay by a fast and slow relaxing component. Within the obtained *T*_1_ and *T*_2_ images, a sample-specific region of interest (ROI) was selected over which the mean (*M*) and standard deviation (SD) were calculated and reported in the concise form of *M*(SD) in the tables contained in the results section.

### 2.7 Upscaling: a Novel Low-Cost 3D Printing unit

A Stepcraft M500 CNC machine (Stepcraft, Menden, Germany) with a customized pneumatic extrusion system was used for 3D extrusion printing of large volume phantoms (>100 mL). A slightly downscaled model of the human brain in mono- and biphasic GM/WM architecture was used as a predefined pattern, with maximum dimensions of 97 mm × 90 mm × 44 mm.

#### 2.7.1 Monophasic Sample without *T*_1_ and *T*_2_ Adjustment

As a proof-of-principle and a well-established control material, an ink based on 3% sodium alginate and 9% methylcellulose (3Alg-9MC), which had been used for the 3D bioprinting of clinically relevant dimensions,^42^ on the commercial bioprinter instrument used above, was used for comparative purposes and compared to the printing behavior of the developed MR-active ink 3Alg-0.8Agar-6Car. This 3Alg-9MC material was prepared as described previously.^42^ However, after the conventionally applied cross-linking with 100 mM CaCl_2_, this construct is not stable enough in the *z*-dimension (height). Therefore, the 3Alg-0.8Agar-6Car ink-based construct was cross-linked using an excess amount of 1 M CaCl_2_ and stored in a humidified atmosphere at 4 °C without an additional storage solution. To determine the stability of the fabricated hydrogel phantom, we regularly documented the height over a course of 100 days using a digital caliper gauge (L826.1, 0–50 mm; Roth, Germany).

#### 2.7.2 Biphasic Phantom with GM/WM-Distinguished Relaxation Times *T*_1_ and *T*_2_

A biphasic phantom consisting of GM and WM zone with anthropomorphic brain geometry was fabricated with dimensions of approximately 100 mm × 60 mm × 44 mm using the customized pneumatic extrusion system equipped with two valves, cartridges and 840 μm-nozzles based on the Stepcraft CNC machine. As respective GM-/WM-mimicking ink materials, 3Alg-0.5Agar-6Car (for GM) and 3Alg-0.8Agar-6Car (280) (for WM), were selected. Ten millimolar CaCl_2_ was added as a covering solution representing the surrounding CSF, which makes the final phantom a triphasic system in total. Measurements were performed 48 h after completed fabrication. As described for the phantom samples of lower volume above, *T*_1_ and *T*_2_ measurements for the following processing, analysis, and graphical representation were operated and performed at PTB.

The design of all different phantom samples applied for the path of analysis of the study are summarized in Table 2.

**Table 2:** Respective Sample Designs for the Workflow of Material Development and Sample Characterization Steps in This Project.

	sample design	max. dimension	AM hardware^a^	anatomical shape	experiments/analysis
ink printability tests	square, 2 layers	10 mm	BS		light microscopy
precharacterization	small	cylinder *d* = 14 mm, *z* = 30 mm	BS		*T*_1_, *T*_2_ ink vs gel
					Car/Agar/GdCl_3_ impact
					long-term stability *T*_1_, *T*_2_ (25 months)
microstructure, GdCl_3_ distribution	disk	cylinder: *d* = 12 mm, *z* = 7 mm	BS		SEM/EDX
monophasic anthropomorphic phantoms	GM/WM slab	57 mm × 39 mm × 11 mm (*x*, *y*, *z*)	BS	GM/WM combined/fused	*T*_1_, *T*_2_
biphasic anthropomorphic phantoms	GM/WM slab	57 mm × 39 mm × 11 mm (*x*, *y*, *z*)	BS	GM/WM defined	*T*_1_, *T*_2_
upscaling demonstrators monophasic	brain	97 mm × 90 mm × 44 mm	SC		height stability (100 days)
upscaling demonstrators bi(tri)phasic	GM/WM brain + CSF	95 mm × 60 mm × 55 mm + 350 mL 10 mM CaCl_2_	SC		*T*_1_, *T*_2_

a BS = BioScaffolder 3.1; SC = Stepcraft machine, customized.

### 2.8 Statistical Analysis

Data analysis and statistical evaluation was conducted using *GraphPad Prism* (version 8; GraphPad Software, CA, USA).

## 3 Results and Discussion

### 3.1 Rheological Characterization of Pastelike Material Blends for Ink Selection

**Figure 1:**
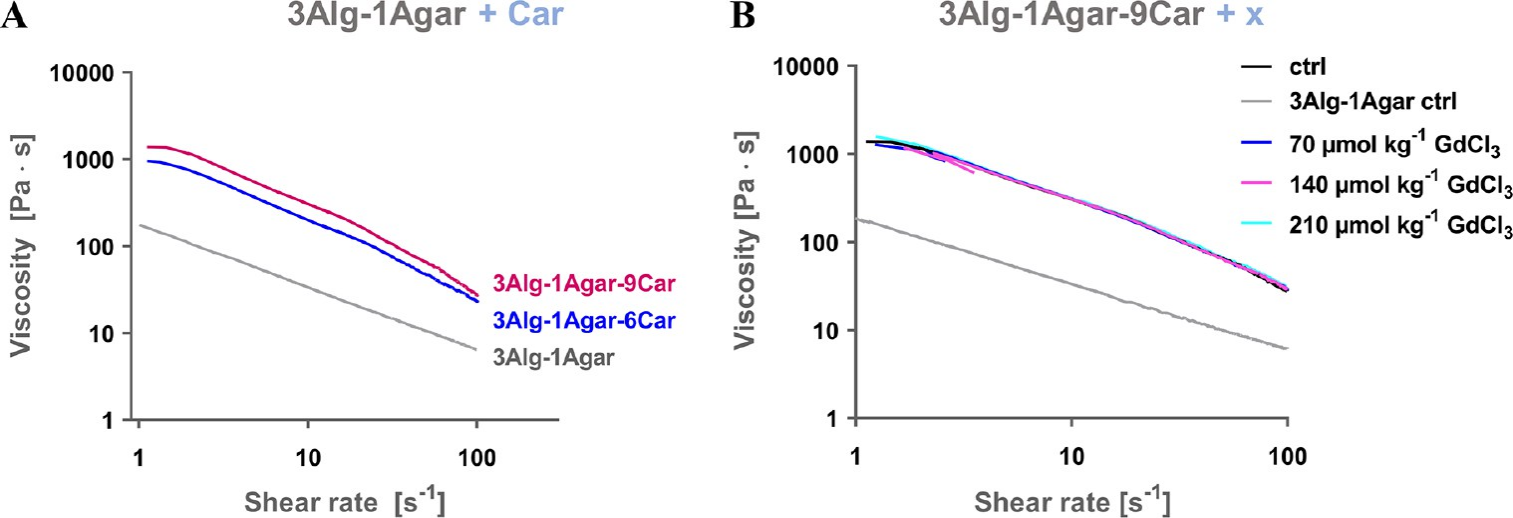
Shear thinning rheological behavior of the suggested ink base material 3Alg-1Agar: (A) with and without addition of carrageenan (Car; 6%/9%). Car is needed for increasing the viscosity of the 3Alg-1Agar ink. (B) Shear thinning behavior of 3Alg-1Agar-9Car with different concentrations of GdCl_3_ (0; 70; 140; 210 μmol kg^–1^) in a shear ramp experiment with increasing shear rates from 1 to 100 s^–1^.

In order to determine which material blends based on the suggested biopolymers alginate, agarose, and ι-carrageenan are suitable as 3D printable inks and can be considered for phantom generation, we need to evaluate the viscoelastic properties. In a shear ramp test increasing the shear rate from 1 to 100 s^–1^, the viscosity of the material for this increasing shear rate is measured. Representative curves are shown in Figure 1. For the blend of alginate and agarose, already a favorable shear thinning behavior was detected, which is essential for deposition of stable strands. The curves for addition of 6 and 9% ι-carrageenan proved that the viscosity can be enhanced successfully by carrageenan addition in the observed range. The effect of 6% addition compared to the carrageenan-free paste was quite drastic (approximately 10-fold increase), while the addition of 9% led to a slight further raise of viscosity. Applying the 3Alg-1Agar-9Car ink in combination with different concentrations of paramagnetic salt ions of GdCl_3_ (70–210 μmol kg^–1^) did not result in a significant effect on the viscosity of the material. The effect of carrageenan addition on viscosity was confirmed for GdCl_3_-containing blends as well (Figure S1).

**Figure 2:**
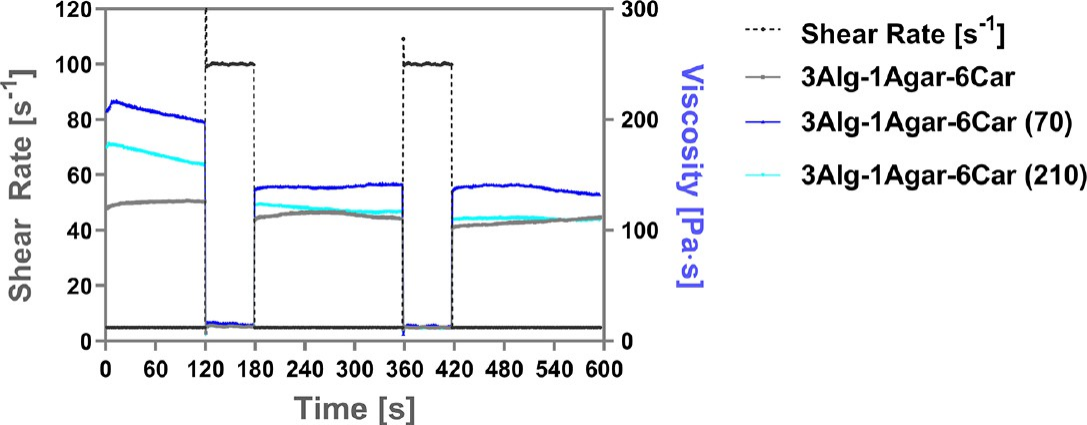
Shear recovery test to evaluate another essential viscoelastic requirement for printable Alg-Agar(-Car) blends, with time-wise defined shear rate intervals of 5 and 100 s^–1^.

For the suggested ink composition with 6% carrageenan, we investigated the shear recovery behavior for GdCl_3_-free and GdCl_3_-supported blends (Figure 2). In this case, before applying higher shear rates, viscosity was enhanced for both GdCl_3_-supported inks with 70 and 210 μmol kg^–1^. However, after applying a high shear rate of 100 s^–1^ for 60 s, the viscosity values were reduced in a rather similar fashion, with a slightly higher viscosity for 3Alg-1Agar-6Car supplemented with 70 μmol kg^–1^ GdCl_3_, in the range of 120 Pa s. In general, the blends were not able to fully reach the initial viscosity after applying a shear rate of 100 s^–1^. However, after a second round of higher shear rate, the viscosity remained rather stable for all the blends.

Such rheological analysis in combination with mass flow data can be applied to calculate the shear stress experienced inside the nozzle by computational models if needed.^46^ Further rheological analysis revealed that the shear thinning properties and the required viscosity for printing can also be adjusted via adaptation of the agarose (0.8–1.2%) and carrageenan (6–9%) content. The ranges for adjustments of viscosity here, however, might be rather marginal (Figure S2). For long-term storage of phantoms, *ProClin150* was added as a biocide later, which in the applied concentration of 0.1% did not affect the viscoelastic properties significantly (data not shown).

### 3.2 Printability and Shape Fidelity of Deposited Strands

**Figure 3:**
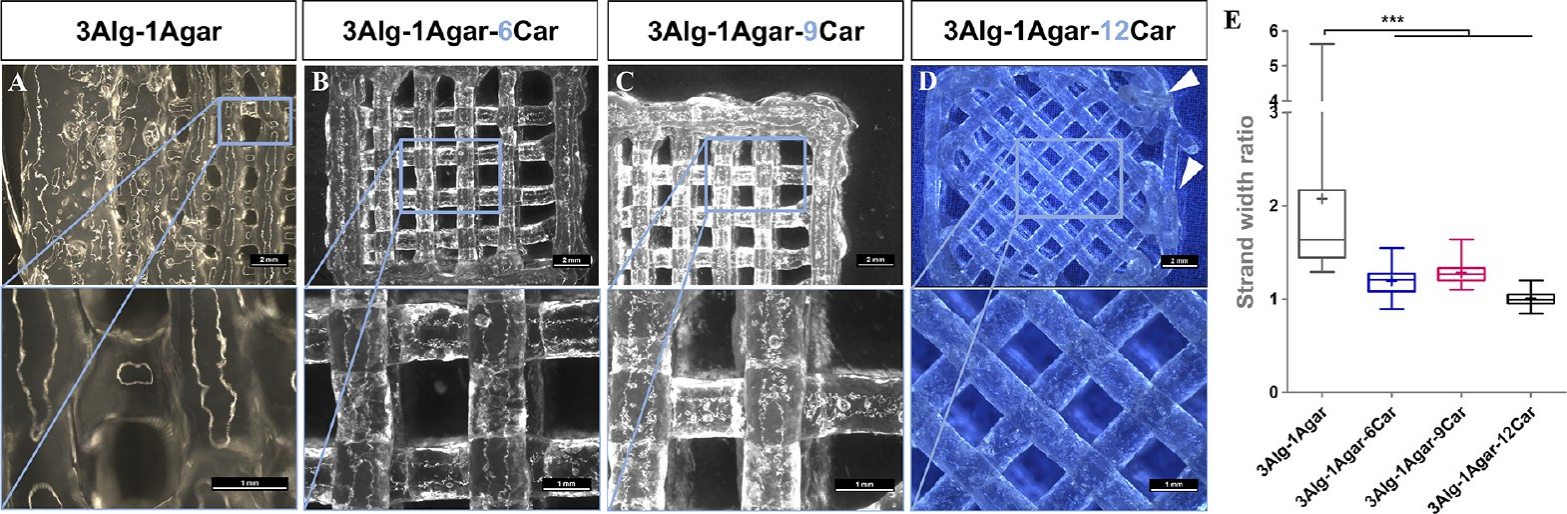
Printability of the suggested MR-active inks and necessity of carrageenan as a thickener while generating an open macroporous, square-shaped structure, to observe the strand-to-strand adhesion, orientation and stability of single strands (necessary to achieve high shape fidelity and volumetric dimensions later). Top row: scale bars = 2 mm, bottom row: scale bars = 1 mm. (E) Quantification of relative strand width ratio, *n* = 20, *** *p* < 0.001.

In order to evaluate and compare the printability and strand deposition behavior, we used simple square shapes with open macropores as known from bioprinting approaches^37,47^ as model objects. Four layers of material were printed before the test phantoms were imaged by light microscopy. The impact of different concentrations of ι-carrageenan (Car) as thickening agent is demonstrated in Figure 3. Without the addition of Car (0%), the 3Alg-1Agar strands deliquesce rapidly and fuse to each other, with a drastic degradation of shape fidelity. As soon as 6% Car is added, an adequate shape fidelity (stable strand geometry without deliquescing) can be achieved. The same is true for the addition of 9% Car. This confirms that Car addition is needed for suitable printing properties. However, when the Car concentration (12%) is drastically increased, the strands can be deposited but the strand-to-strand and layer-to-layer adhesion, especially in the *z*-direction, cannot be ensured (Figure 3, right, arrows). This composition cannot be used for phantom fabrication and has been excluded from further analysis. For the phantom printing, such open porous structures as used here only for visualization of strand shape fidelity (infill distance 2.3 mm) will not be preferred for the later course of the project in order to reach the aim of homogeneous signal contrasts in defined compartments. Quantification confirmed the finding that the addition of Car (6–12%) results in an improved strand width ratio (Figure 3E).

**Figure 4:**
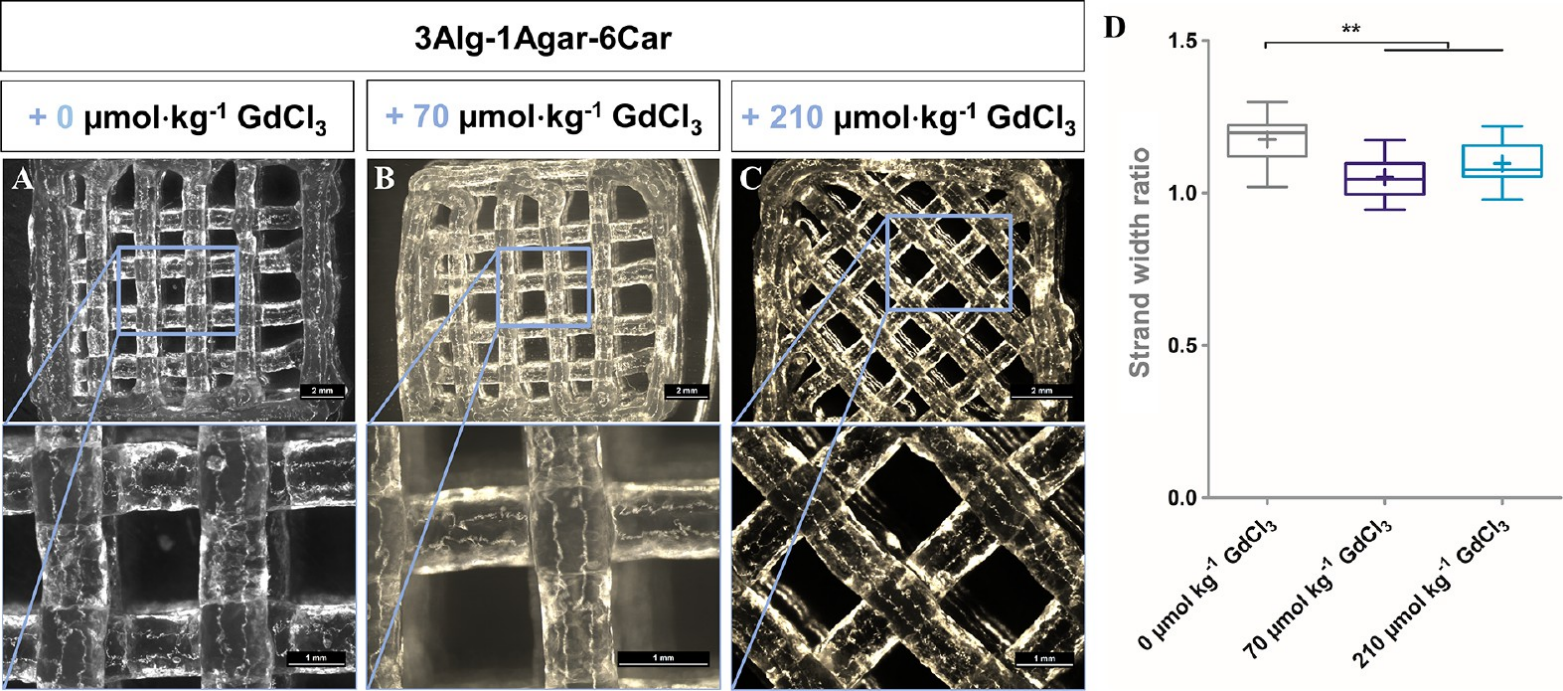
Printability of the suggested MR-active inks without/with GdCl_3_ supplementation in μmol kg^–1^. Top row: scale bars = 2 mm, bottom row: scale bars = 1 mm. (D) Quantification of relative strand width ratio, *n* = 20, ** *p* < 0.01.

In the same manner, the impact of GdCl_3_ addition to strand deposition was observed. After adding different concentrations of GdCl_3_ to trigger the relaxation time *T*_1_ in similar concentration ranges as suggested recently,^21,23^ no adverse impact on printability and shape formation was detected (Figure 4) for 0, 70, and 210 μmol kg^–1^. As the addition of GdCl_3_ appeared to have a slight effect on the shape fidelity (reducing the strand width ratio to values closer to 1.0), too, for the later course of the study, the same volume of H_2_O as in the GdCl_3_ solutions was added to the otherwise plain Alg-Agar-Car control gels (no GdCl_3_) to ensure a more reliable comparison between the inks.

Different angles and printing patterns between the filling strands or between an outline or the infill pattern, as demonstrated for the 3Alg-1Agar-12Car ink in Figure 3 without GdCl_3_ and for the 3Alg-1Agar-6Car ink with a GdCl_3_ concentration of 210 μmol kg^–1^ in Figure 4, can be a solution in the further course of the study to ensure closing of the open pores in between strands and in order to print more homogeneously.

As an aim of the study, clinically relevant dimensions will need to be reached. In order to achieve a sufficient resolution with an already extended printing time for large dimensions while avoiding overengineering, we chose an inner needle diameter of 840 μm throughout the project, although a higher resolution could be achieved with the suggested materials.

The analysis of the strand diameter and of potential hydrogel swelling phenomena in open-porous hydrogel structures after printing was performed using collected light-microscopical images. The strand diameter was quantified during incubation in different storage environments, considering pre/post cross-linking and a course of 8 days for 3D printed open-porous hydrogels based on 3Alg-1Agar-9Car as an exemplary ink; the data demonstrated the suitability of the ink due to its low extent of swelling (Figure S3). The volume of aqueous GdCl_3_ solutions for supplementing the ink with different concentrations was constant, independent of the final GdCl_3_ concentration. The concentration was adjusted by the concentration of the stock solution. The solubility of GdCl_3_ was ensured in the applied concentration ranges. Therefore, possible observed phenomena (on printing behavior, rheological properties, relaxation times) would be only due to the effects of GdCl_3_ itself rather than by different amount of water added to the inks during the preparation process as described above.

### 3.3 Microstructure of the Phantom Hydrogels and GdCl_3_ Distribution

**Figure 5:**
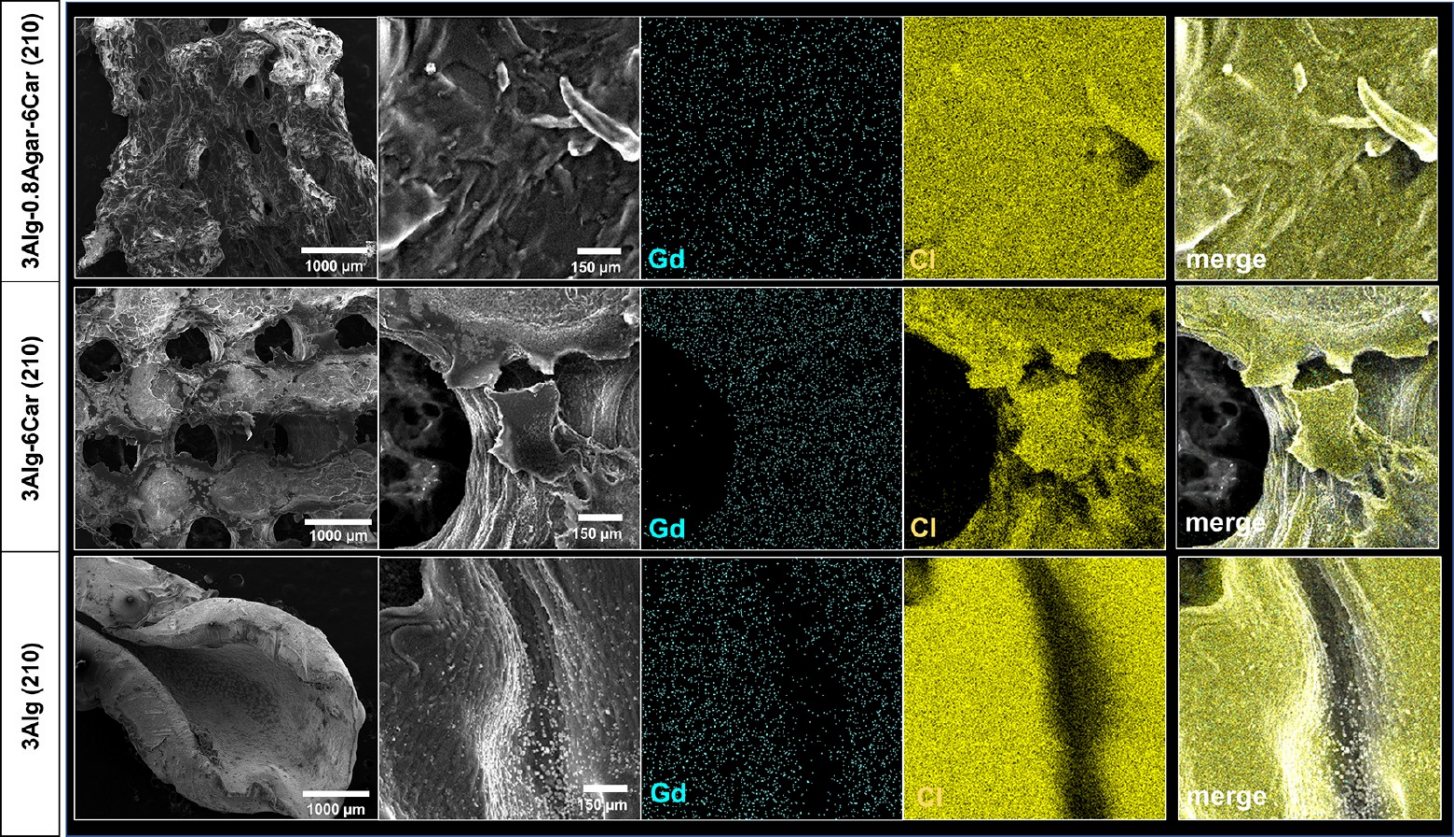
SEM-based microstructure images of phantom samples consisting of 3Alg (bottom), 3Alg-6Car (middle) and 3Alg-0.8Agar-6Car (top row) with 210 μmol kg^–1^ GdCl_3_ each (sample overview left column) and EDX-based element analysis for Gd and Cl. Scale bars in the first column represent 1000 μm, scale bars in the second column represent 150 μm.

By SEM, we investigated the microstructure of the material without the need for a drying and sputtering process. Furthermore, EDX analysis allowed us to distinguish different elements as part of the ink components of our MR-active hydrogel. The sample was homogeneously cross-linked through the alginate. By addition of agarose and Car, the rather smooth plain alginate surface became rougher and more rugged. A homogeneous distribution of Gd within the 3D printed strand was detected (Figure 5), although even the concentration of 210 μmol kg^–1^ (as the highest concentration applied in most experiments of the study) resulted in a rather disperse signal throughout the tested samples. The higher polymer content that is needed for suitable printability, after addition of 0.8% agarose and 6% Car, did still allow a homogeneous distribution of Gd.

### 3.4 Impact of GdCl_3_ Content and Alginate Cross-Linking on *T*_1_ and*T*_2_

**Figure 6:**
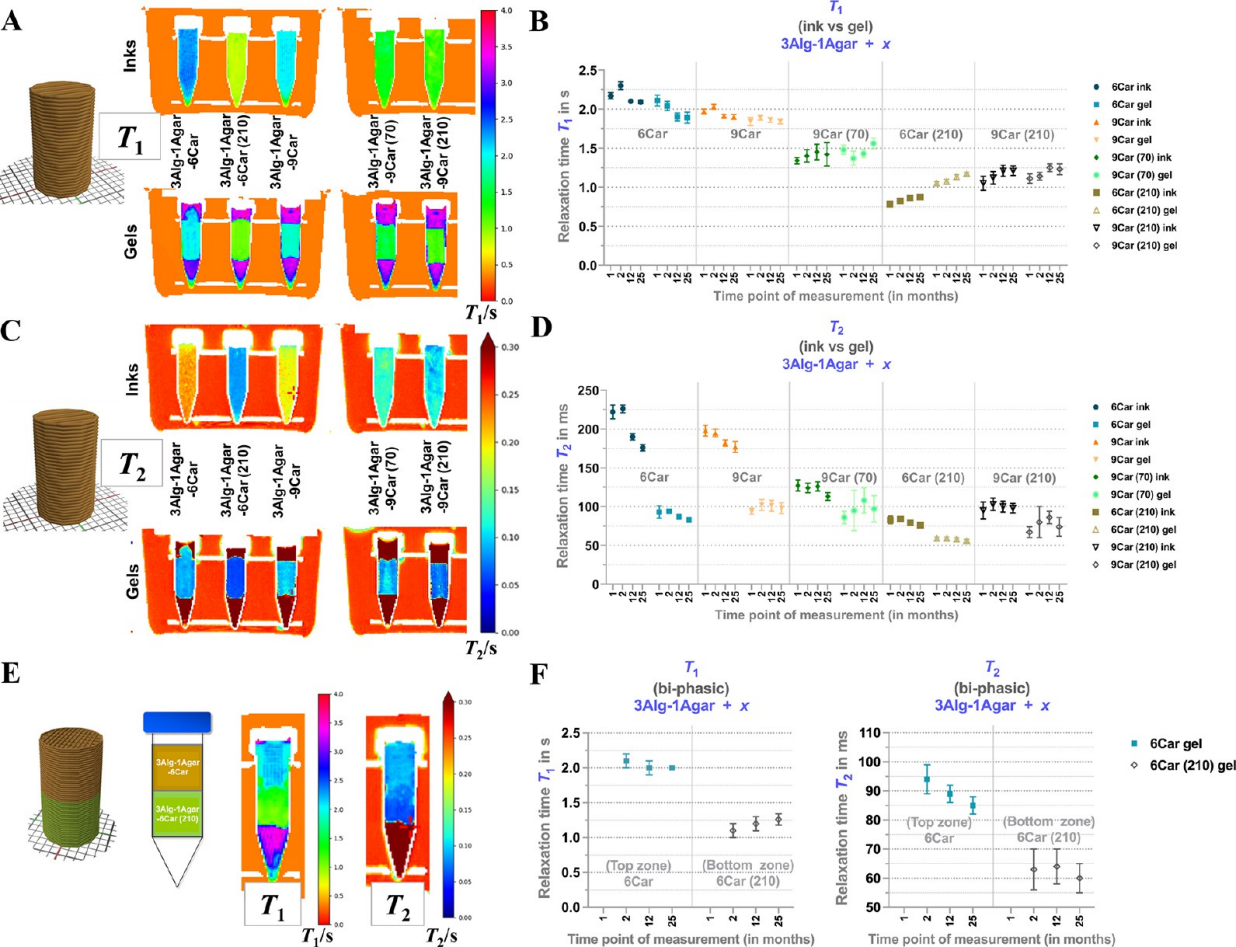
*T*_1_ and *T*_2_ signals of MR-active inks based on 3Alg-1Agar prior to printing (“inks”) and after postfabrication cross-linking (1 M CaCl_2_ 20 min, “gels”) in small phantom samples in 5 mL tubes. (A, B) Visualizing *T*_1_ after 1 month, and graphing *T*_1_ after 1, 2, 12, and 25 months postfabrication in monophasic phantoms (*T*_1_ in s). (C, D) Visualizing *T*_2_ (in s) after 1 month, and graphing *T*_2_ (in ms) after 1, 2, 12 and 25 months postfabrication in monophasic phantoms. (E, F) Visualizing *T*_1_ and*T*_2_ (both in s) after 2 months, and graphing *T*_1_ (in s) and *T*_2_ (in ms) after 2, 12, and 25 months postfabrication in biphasic phantoms based on 3Alg-1Agar-6Car as the top compartment 3Alg-1Agar-6Car (210) as the bottom compartment.

For the initial characterization of *T*_1_ and *T*_2_ of printable inks and printed monophasic phantoms, we chose cylindrical samples of small dimensions (≤5 mL) that fit into 5 mL Eppendorf tubes. In this manner, the non-cross-linked inks (Figure 6, “Inks”) and the resulting hydrogels (Figure 6, “Gels”) were prepared and arranged in respective racks for measurements (Figure S4). Given the fact that these material compositions can be used as printable inks (Figures 3 and 4), we wanted to know how the relaxation times can be triggered by the ink composition based on ι-carrageenan (in %) and GdCl_3_ (in μmol kg^–1^) concentrations, and preliminarily, how different ink compositions can form a *T*_1_-, *T*_2_-distinguishable interface of two compartments. The stability of the obtained values was demonstrated over a course of 25 months (Figures S5 and S6). Initially, it was evaluated to what extent the forming network by CaCl_2_-mediated cross-linking of the alginate chains affects the adjustment of relaxation times: *T*_1_ appeared rather stable in response to cross-linking, whereas *T*_2_ was strongly reduced by the cross-linking process (Figure 6A, B), i.e., this needs to be considered in the further course of the study. As a second step, we examined how the ink components of Car and GdCl_3_ affect *T*_1_ and *T*_2_: In general, with the suggested compositions, *T*_1_ was adjustable in the range of approximately 1–2 s, which almost covers the range of gray and white matter;^48^ however, a bit more tuning of the relaxation times to lower values might be needed for the white matter. *T*_2_ was adjustable^49^ in the range of approximately 50–110 ms, which can cover the range of gray and white matter^50^ too. For GdCl_3_-free samples, *T*_1_ was slightly reduced with increasing carrageenan content (6–9%), while both *T*_1_ and *T*_2_ appeared slightly reduced after longer storage times.

For GdCl_3_-containing samples, the impact of carrageenan content on *T*_1_ or *T*_2_ was not significant. Although a gel with 9% of carrageenan was expected to be more stable in a mechanical manner and might allow a lower extent of ion diffusion without a physical barrier, we observed that homogeneity was reduced as printability was not as good as with 6% of Car. Therefore, for the following steps of ink optimization, we decided to restrict the Car content to 6% and investigate the effects of agarose as possible *T*_2_-modifier. As suggested by former studies on agarose^16,23^ and agar^19^ as a main (gelling) component in thermo-reversible but not printable gels, agarose has typically been proposed as a factor to adjust *T*_2_.^23^

In the biphasic sample of 3Alg-1Agar-6Car (+210 μmol kg^–1^ GdCl_3_) and 3Alg-1Agar-6Car (w/o GdCl_3_), clearly distinguishable values for *T*_1_ and *T*_2_ were detected (Figure 6E), with contrast-to-noise ratios of 10 and 4 for *T*_1_ and *T*_2_, respectively (using the values obtained 2 months postfabrication). These *T*_1_ and *T*_2_ values remained rather stable over time, only *T*_2_ of the Gd-free zone was slightly reduced after 25 months (Figure 6F).

All 3D printed samples with 9% carrageenan content showed a clear second *T*_2_-component making the fit-result sensitive on how to weight the intensities from the different TE-images. However, even with the more sample points in TE as acquired after 12 months, a double exponential fit does not converge for all pixels to comparable values. It seemed that a long *T*_2_ component in the range of 250 to 350 ms exists within the 3D printed and cross-linked gel samples. Consequently, by respecting this second longer *T*_2_, the values of the shorter component have become even smaller, e.g. 60 ms for 3Alg-1Agar-9Car (70) and 50 ms for 3Alg-1Agar-9Car (210).

The long (∼0.3 s) *T*_2_ for the storage liquid (0.1 M CaCl_2_) surrounding the gel samples indicated a low salt concentration. This can be due to the gels absorbing free ions or can indicate that the high concentration gradient from gel to solution remains rather stable over time.

### 3.5 Impact of GdCl_3_ and Agarose Content and Cross-Linking Ion Concentration on *T*_1_ and *T*_2_

In this next experiment, the impact of the agarose on relaxation times with and without the addition of 70 μmol kg^–1^ GdCl_3_ was investigated on cross-linked samples of the identical small volume (<5 mL). Four different concentrations of agarose (0, 0.5, 0.8, and 1.0%) in combination with two different contents of GdCl_3_ (0 μmol kg^–1^, 70 μmol kg^–1^) were considered. For the long-term stability of samples, for larger dimensions in nonsealed containers mainly, fungicide and bactericide need to be added to avoid contamination and respective degradation. Therefore, 0.1% *ProClin150* was also added to the inks used here prior to printing.

**Figure 7:**
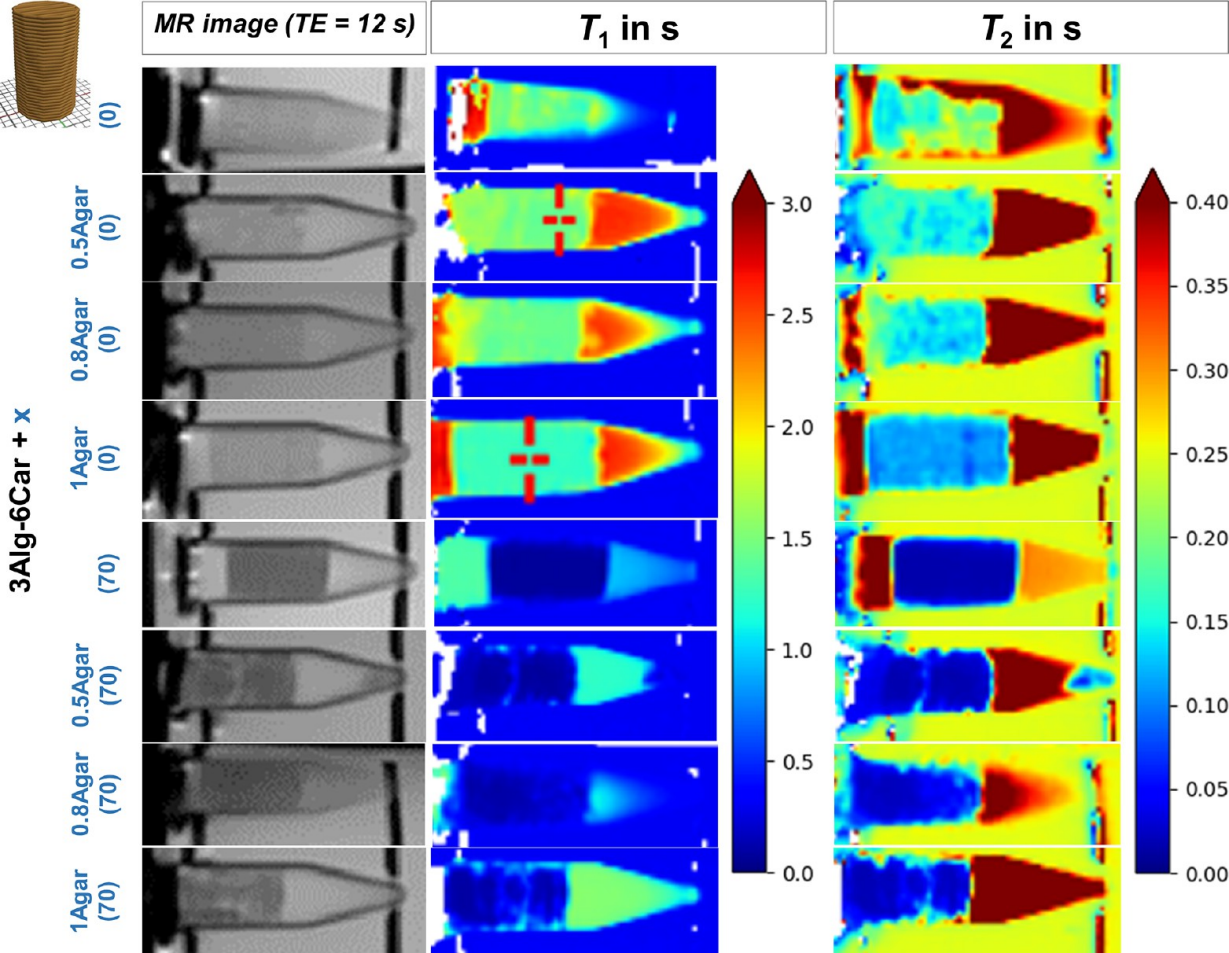
*T*_1_ and *T*_2_ measurements of MR-active monophasic gels printed as small phantom samples and placed into 5 mL tubes after cross-linking with 1 M CaCl_2_. Visualizing *T*_1_ and *T*_2_ (both in s) in monophasic phantoms based on 3Alg-6Car with different concentrations of agarose (0, 0.5, 0.8, 1.0%) and GdCl_3_ (0, 70 μmol kg^–1^).

Visualization of *T*_1_ and *T*_2_ is shown in Figure 7, and mean values detected for the samples are listed in Table 3. Results revealed that by agarose and GdCl_3_ admixture in 3Alg-6Car gels, *T*_1_ was adjustable (1.5 or 0.2 s) in a range up to 2 s, whereas *T*_2_ was adjustable between 0.02 and 0.15 s. For *T*_1_, without GdCl_3_, no significant effect by agarose (0–1%) was detected, maybe a slight decrease in response to higher concentration. In general, for this set of Eppendorf-shaped samples, the *T*_1_ values were much lower compared to the first experiment. The printing conditions and homogeneity of the internal structure play a major role here. After addition of GdCl_3_ (70 μmol kg^–1^), the addition of more agarose (1%) led to a slight shift toward higher *T*_1_ but reduced the homogeneity of the phantom and the signal. For *T*_2_, without GdCl_3_, addition of agarose in the observed range led to reduction of *T*_2_ (more clearly detected with respect to the second fast relaxing *T*_2_ component), while after addition of GdCl_3_ (70 μmol kg^–1^), no significant effect by agarose on *T*_2_ was noticed. However, the slow relaxing *T*_2_ component is almost completely suppressed by the GdCl_3_.

**Table 3:** *T*_1_ and *T*_2_ Values Obtained for Measurements of Eppendorf Samples (in Figures 7 and 8)^a^

				double exp.		
	GdCl_3_(μmol kg^–1^)	cross-link *c*(CaCl) in M	single exp. *T*_2_ (±SD) (ms)	*T*_2_ slow (±SD) (ms)	*T*_2_ fast (±SD) (ms)	*T*_1_ (±SD) (s)
	agarose impact					
3Alg-6Car	0	1	200 (50)	250 (50)		1.55 (9)
3Alg-0.5Agar-6Car	0	1	150 (10)	170 (10)	[14 (6)]	1.44 (4)
3Alg-0.8Agar-6Car	0	1	150 (10)	180 (10)	[18 (3)]	1.45 (3)
3Alg-1Agar-6Car	0	1	140 (10)	150 (10)	[16 (3)]	1.26 (2)
	agarose impact (+Gd)					
3Alg-6Car	70	1	19 (2)		16 (1)	0.07 (1)
3Alg-0.5Agar-6Car	70	1	26 (11)		17 (2)	0.10 (2)
3Alg-0.8Agar-6Car	70	1	25 (10)	[270 (70)]	20 (3)	0.14 (4)
3Alg-1Agar-6Car	70	1	33 (13)	[260 (90)]	18 (2)	0.15 (6)
	cross-link impact (+Gd)					
3Alg-1Agar-6Car	70	0.1	50 (18)	[150 (50)]	20 (2)	0.16 (3)
3Alg-1Agar-6Car	70	0.5	29 (15)	[400 (90)]	16 (2)	0.18 (8)
3Alg-1Agar-6Car	70	1	33 (13)	[260 (90)]	18 (2)	0.15 (6)

a For the double exponential fit results, the relaxation times for the component with the much smaller contribution is stated in square brackets or not stated in cases where the fit resulted in an SD in the range of the mean value.

Therefore, the main factor tuning both *T*_1_ and *T*_2_ remains the addition of paramagnetic GdCl_3_ salt. This means that the amount of agarose inside the suggested inks can be adjusted more flexibly with respect to the printability without a major effect on relaxation times. This can be helpful since those initial experiments also demonstrated that a reduced agarose content can lead to better homogeneity.

**Figure 8:**
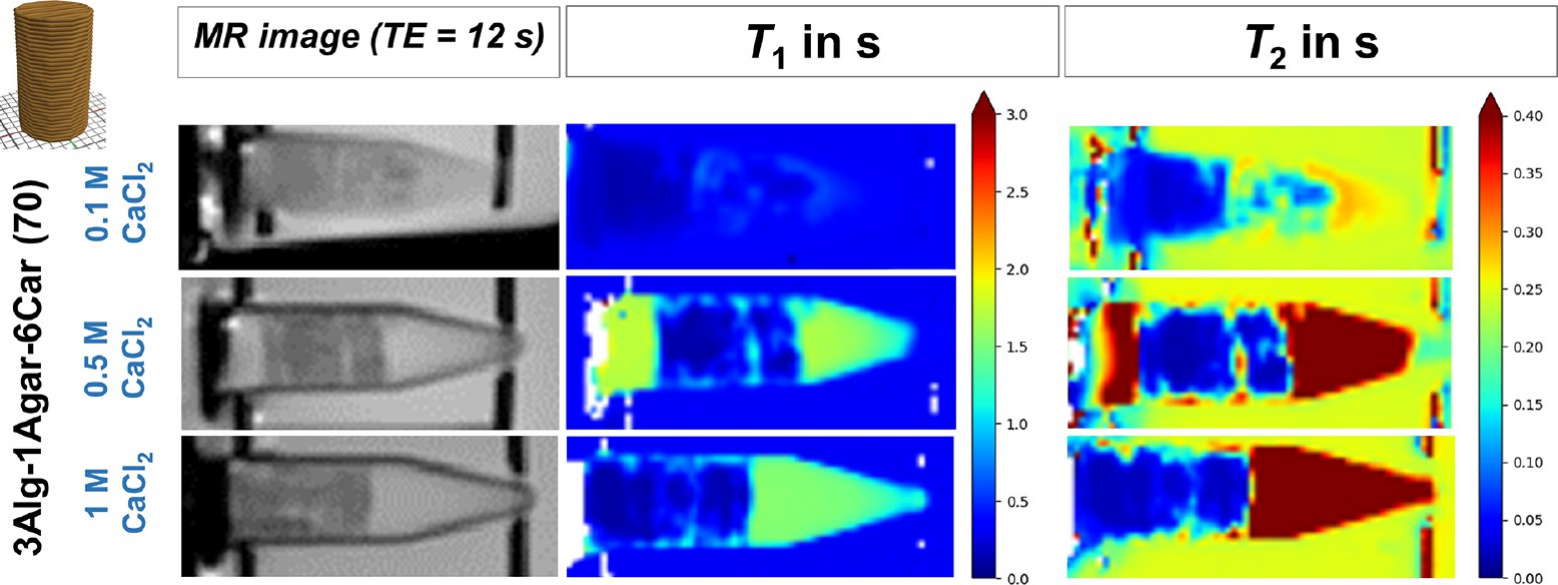
*T*_1_ and *T*_2_ measurements in dependence of cross-linking solution CaCl_2_ concentration (0.1, 0.5, 1.0 M CaCl_2_). *T*_1_ and *T*_2_ images of MR-active monophasic gels as small phantom samples in 5 mL tubes after cross-linking with different solutions. Visualizing *T*_1_ and *T*_2_ (both in s) in monophasic phantoms based on 3Alg-1Agar-6Car.

Besides the ink composition, the cross-linking degree affects the density and the microstructure of the biopolymer network based on alginate. We expected that this degree of cross-linking mediated by the CaCl_2_ concentration in the external cross-linking solution can play a role for the relaxation times as well. As illustrated in Figure 8, the cross-linking concentration (0.1, 0.5, 1.0 M) had no or only minor effects on *T*_1_ or *T*_2_. However, for sample integrity during handling, for homogeneity and for ensuring a thorough cross-linking of the entire gel, 1 M CaCl_2_ was needed and selected for the further steps (Figure 8). Both *T*_1_ and *T*_2_ of the surrounding 10 mM CaCl_2_ within the Eppendorf tubes were found longer for higher CaCl_2_ cross-linking concentrations which is due to diffusion of gel components into the surrounding solution. For both 0.5 and 1 M CaCl_2_, this effect of diffusion of agarose and especially GdCl_3_ seems to be reduced by a more stable cross-linking through those higher calcium concentrations.

### 3.6 3D Plotting of Monophasic Anthropomorphic 3D Structures with Adjusted Relaxation Times

**Figure 9:**
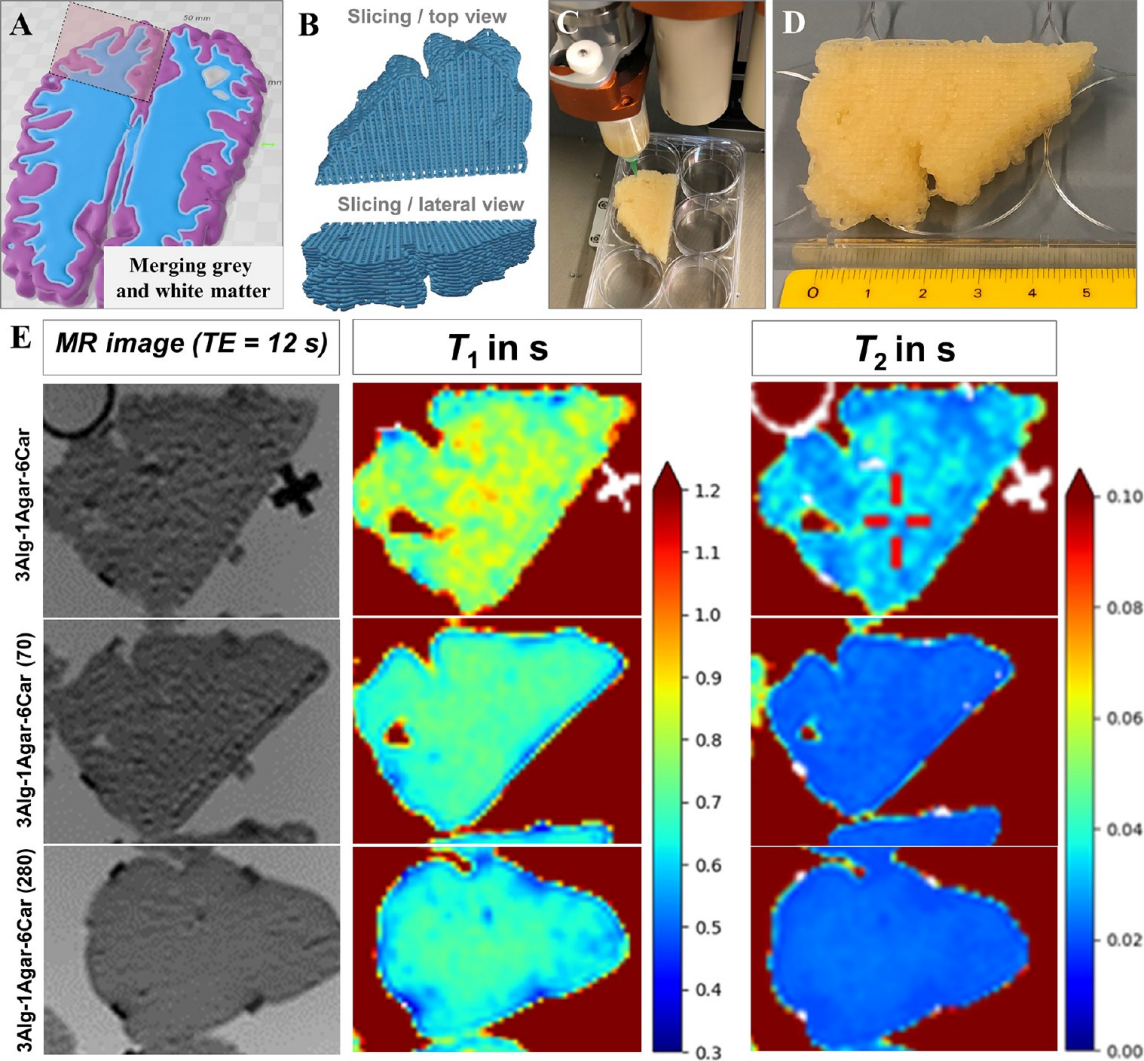
(A) Construction, (B) slicing, and (C) 3D printing of a monophasic anthropomorphic phantom. (D) Photograph of one exemplary sample prior to cross-linking, scale bar in cm. (E) MR image and quantitative determination of relaxation times *T*_1_ and *T*_2_ (both in s) of 3D printed monophasic anthropomorphic phantoms based on 3Alg-1Agar-6Car with 0, 70, and 280 μmol kg^–1^ GdCl_3_.

The reported findings from the preliminary work on inks and cross-linked hydrogel samples of a rather small volume (<5 mL) was transferred to anthropomorphic geometries by virtually fusing GM and WM in a slab of a human brain model (Figure 9A) to one common region (section with maximum dimensions of *x* × *y* × *z* = 57 mm × 39 mm × 11 mm). This combined GM/WM geometry was then translated into a printable G-code, in this case with a 90° layer-to-layer shift of deposited strands (Figure 9B), in order to be printed with the BioScaffolder 3.1 (Figure 9C, D). After *T*_1_ and *T*_2_ measurements, the results were summarized for three different 3D printed phantom samples of 3Alg-1Agar-6Car, 3Alg-1Agar-6Car (70), and an extended GdCl_3_-concentration in 3Alg-1Agar-6Car (280). In this experiment, printability appeared as slightly decreased after the addition of GdCl_3_, which was not consistent with the observations on the rheological properties above. Later, printing parameters were adjusted and optimized accordingly for GdCl_3_-containing inks.

Diffusion of the GdCl_3_ and materials into the surrounding 0.1 M CaCl_2_ storage solution at first appeared as rather weak, so that the ink-adjusted *T*_1_ and *T*_2_ were clearly detectable. The values remained rather stable over time. *T*_1_ and *T*_2_ were adjustable by the GdCl_3_ content in the range of approximately 680–710 ms for *T*_1_ and for *T*_2_ in the range of 20–30 ms (Figure 9, Table 4). The general shape fidelity of the anthropomorphic geometry was maintained. However, the high agarose content of 1% as shown for the developed inks above (Figures 1–4) did not necessarily lead to homogeneous printing results regarding the internal strand-to-strand structure (e.g., for 3Alg-1Agar-6Car, GdCl_3_-free). Therefore, the agarose content was reduced for further work on multiphasic phantoms.

**Table 4:** *T*_1_ and*T*_2_ Values Obtained for Measurements of Monophasic Anthropomorphic Phantoms (in Figure 9)

				double exp.		
	GdCl_3_(μmol kg^–1^)	cross-link *c*(CaCl) in M	single exp. *T*_2_ (±SD) (ms)	*T*_2_ slow (±SD) (ms)	*T*_2_ fast (±SD) (ms)	***T***_**1**_ (±SD) (s)
3Alg-1Agar-6Car	0	1	28 (2)	108 (31)	19 (2)	0.68 (3)
3Alg-1Agar-6Car	70	1	23 (1)		23 (1)	0.68 (2)
3Alg-1Agar-6Car	280	1	22 (1)		22 (1)	0.71 (2)

### 3.7 3D Plotting of Biphasic Anthropomorphic 3D Structures with Adjusted Relaxation Times

**Figure 10:**
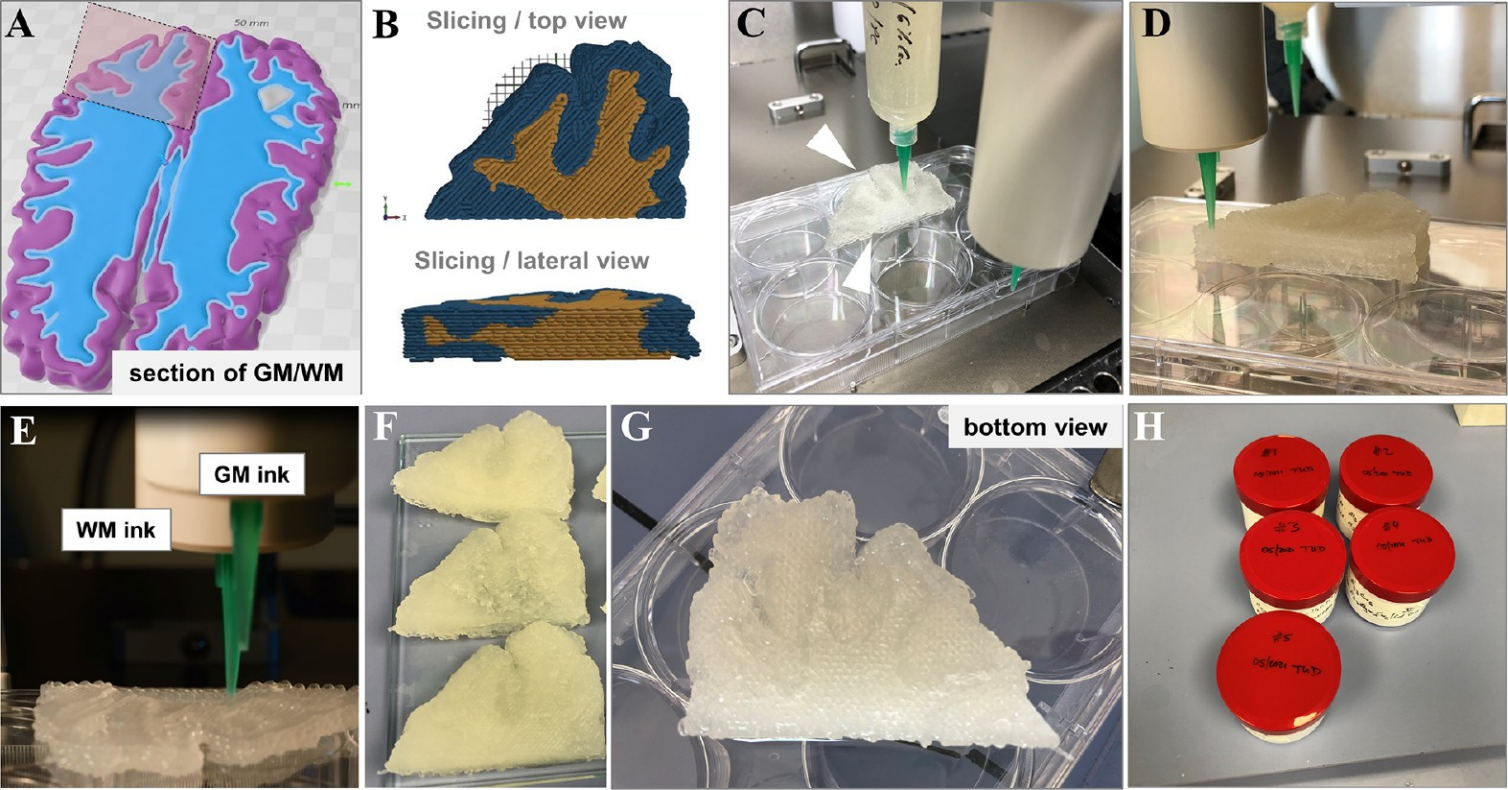
(A) Construction, (B) slicing, and (C–E) 3D printing of a biphasic anthropomorphic phantom; outer dimensions *x* × *y* × *z* = 57 mm × 39 mm × 11 mm. (F, G) Photographs of exemplary sample(s) as top and bottom view postcross-linking; (H) storage containers for sample transport.

For transferring the results from monophasic structures to a biphasic environment, the same brain section as shown in Figure 9 was used, with a maximum size of *x* × *y* × *z* = 57 mm × 39 mm × 11 mm. In this setup (Figure 10A), GM and WM remained separated for the biphasic brain slab construct (Figure 10B). Two materials were processed by the multichannel extrusion printer (Figure 10C). Those biphasic sections were analyzed for their relaxation times *T*_1_ and *T*_2_ and it could be demonstrated that the aim of a spatially defined distribution of both relaxation times *T*_1_ and *T*_2_ was achieved. In all investigated phantoms, the desired sectioned anatomical structure and the biphasic character of the selected slab was visible. After noticing some deviations within the samples through the internal structure, the mean distance between the strand centers was further decreased, while the layer-to-layer angle shift was chosen at 45° instead of 90° (Figure 10B) in order to improve sample homogeneity and avoiding open pores after printing. Despite some minor deformation through an inconsistent mass flow of ink, phantoms were printed with good shape fidelity and kept their shape after cross-linking Figure 10F, G top and bottom view).

**Figure 11:**
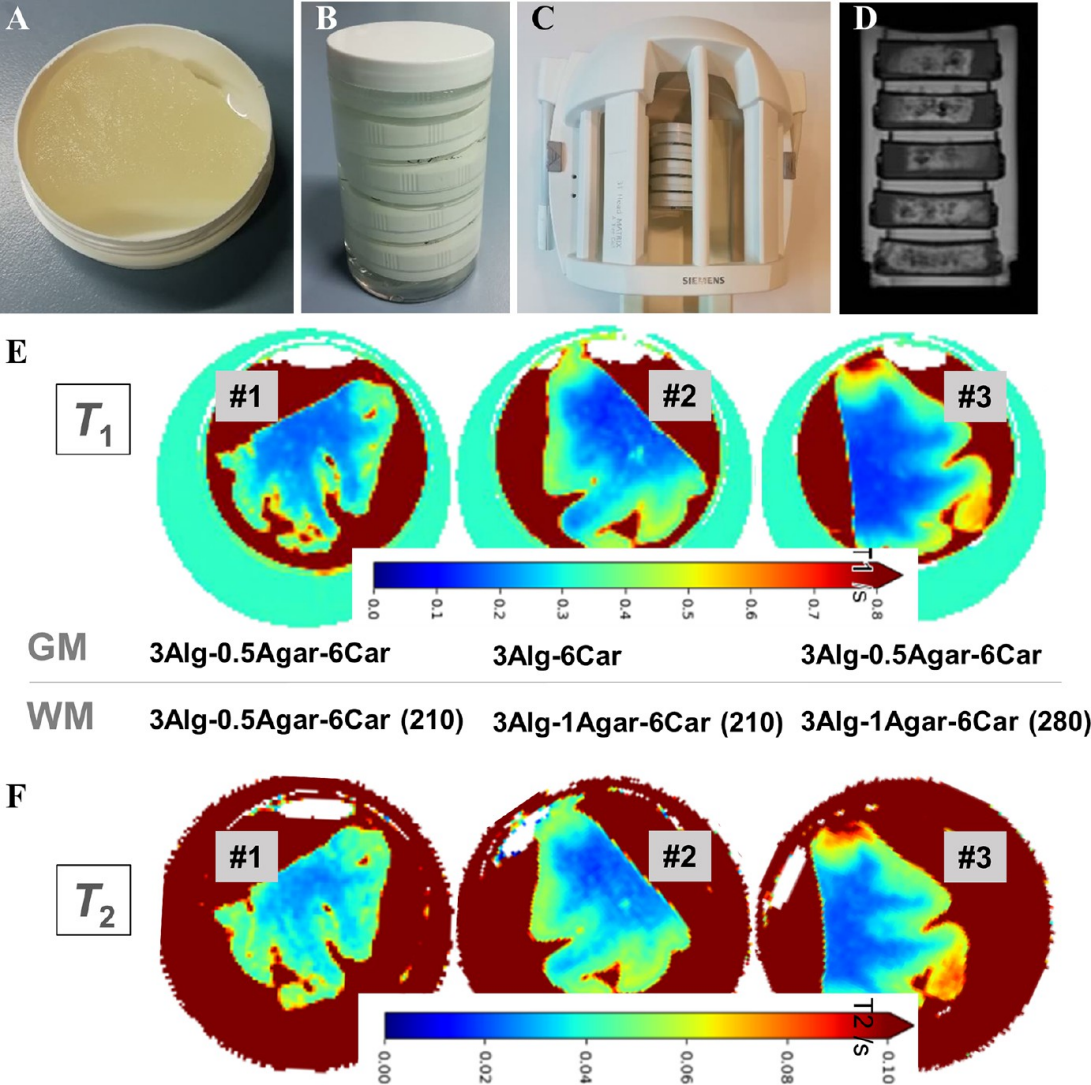
(A–D) Sample preparation, storage containers, and assembly for measurements. (E, F) *T*_1_ and *T*_2_ measurements of 3D printed biphasic anthropomorphic phantoms. Measurements after one month postfabrication (*T*_1_ and *T*_2_ in s) for samples 1–3 with varying GM-WM composition.

For the MR measurements, we placed these samples in plastic jars filled with 0.1 M CaCl_2_ and 0.1% *ProClin150* for fixation of a maximum of five samples in the MR coil (Figure 11A–C). Shape fidelity was maintained throughout sample processing, shipment, and placing inside the measurement containers. The few points with visible inhomogeneities were found in both *T*_1_ and *T*_2_ images (Figure 11E, F), indicating that printing artifacts or material aggregates were responsible for those irregularities rather than diffusion of ions. For *T*_1_ measurements (Figure 11E), the clear effect by the addition of GdCl_3_ to the WM material was confirmed by a decreased *T*_1_. Increasing the GdCl_3_ concentration from 210 to 280 μmol kg^–1^ resulted in an even slightly lower *T*_1_ value.

Equivalently to *T*_1_, the *T*_2_ values of the surrounding CaCl_2_ solution also correlated with the GdCl_3_ concentration of the corresponding samples. Over time, a reduction of *T*_1_ in GM was observed, while *T*_1_ in WM slightly increased. Over the observed 8 months, reduced *T*_2_ values were also detected in GM, while in WM, those values increased slightly. Apparently, an ion exchange or balancing between the two compartments was revealed. Also, for *T*_2_ measurements (Figure 11F), the addition of GdCl_3_ to the WM material led to shorter *T*_2_ times. All *T*_2_ times are well below 100 ms; the aimed values of approximately 90 and 60 ms for GM and WM,^49^ respectively, are not fully reached. Optimization on these biphasic samples will be required. Understanding and controlling of the *T*_1_ and *T*_2_ adjustment and later development will be an important challenge to focus on.

The signal decay within pixels from both liquids (CaCl_2_ solution as well as HEC) showed in a logarithmic scaling an almost perfect linear dependence. However, bending effects were observed for pixels stemming from some of the 3D-printed material. Consequently, when looking to the residual after performing a monoexponential fit for those pixels, the typical structure resulting from a second decay component was visible. However, this effect was too small for a double exponential fit to be converged for all samples. Thus, only results from the monoexponential decay fit are presented for those where the double exponential fit led to very large uncertainties (Table 5).

**Table 5:** *T*_1_ and *T*_2_ Values Obtained for Measurements of Biphasic Anthropomorphic Phantoms (in Figure 11) after 1, 2, and 8 months postfabrication

	GM			WM		
*T*_1_ (s)						
time after fabrication in months	1	2	8	1	2	8
sample 1	0.35 (1)	0.32 (2)	0.22–0.36	0.19 (1)	0.22 (3)	0.23 (3)
sample 2	0.35–0.42	0.27 (2)	0.23 (1)	0.17 (2)	0.22 (2)	0.23–0.37
sample 3	0.37 (2)	0.26–0.36	0.23–0.39	0.145 (10)	0.18 (2)	0.20 (5)
*T*_2_ (s)						
time after fabrication in months	1	2	8	1	2	8
sample 1	44 (2)	43 (1)	41 (2)	27 (2)	31 (1)	34 (2)
sample 2	46 (4)	42 (2)	37 (4)	23 (3)	28 (4)	38 (4)
sample 3	50 (7)	45 (5)	42 (5)	22 (2)	26 (2)	33 (2)

For the presented concepts, further optimization and fine-tuning of the relaxation times will be needed as was shown. However, comparing to phantom materials with a simpler composition, a free adjustment through the three-component biopolymer system (+GdCl_3_ concentration) as presented here is also possible but requires further work for fine-tuning. Yet an important unanswered question remains whether the material selection would allow a specific definition of physiological relaxation times. Furthermore, other factors can play a role in the presented technique: In theory, a stiffness and density gradient could also be induced by altered cross-linking concentrations^51^ which might help to trigger or hinder the diffusion of GdCl_3_. Imagining clinical brain MRI scans, the interface between GM and WM is not expected as fully sharp depending on the MRI scan quality. Therefore, slight amount of ion diffusion can be tolerated. As we were able to show with our Alg-Agar-Car system, no additional material barrier was needed to prevent an early onset of diffusion, so that even after longer storage times (25 months), a contrast in MR images was detectable. Since both GM and WM were based on different material compositions with an identical cross-linking scheme, fusion, and adhesion between the compartments was ensured at the same time.

One common challenge—also for other approaches—is the independent adjustment of *T*_1_ and *T*_2_. In our case, the agarose in the applied concentrations did not have huge effects on *T*_2_, unlike what has been suggested^21^ for other material blends. Therefore, mainly the overall polymer content and the GdCl_3_ concentration were responsible for adjustment of both *T*_1_ and *T*_2_. Over time, the relaxation times in both compartments had converged to each other. However, even after a long storage time, the zones were still distinguishable (Figure 6E, F; Figure S6).

### 3.8 Toward Large-Scale Fabrication in Anatomical Dimensions

Many approaches have been developed for phantom fabrication over the years: Altermatt and colleagues presented a casted biphasic phantom based on agar and MnCl_2_ with silicone molds mimicking the brain folding pattern,^19^ while Gopalan and co-workers demonstrated the possibility of a partly (GM-) printed biphasic phantom based on agar and MnCl_2_ as well^18,26^ and later suggested breaking down the brain pattern into phantom slices.^18^ With nonprintable hydrogels fully characterized for *T*_1_ or *T*_2_ impacts before,^16,21,23^ reproducing real GM and WM relaxation times was better achieved compared to our approach.

**Figure 12:**
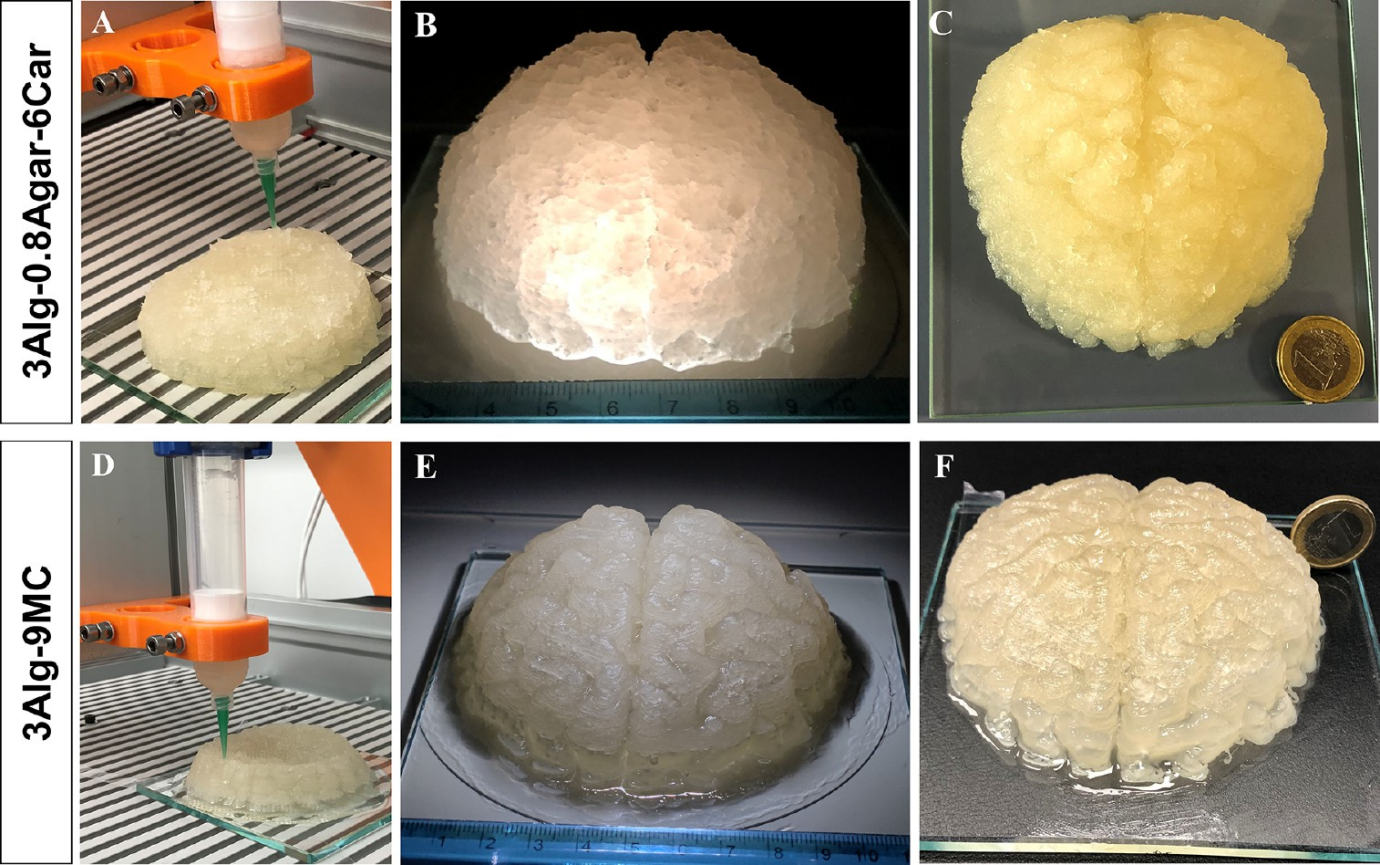
Upscaling toward volumetric anthropomorphic dimensions. (A) A 3Alg-0.8Agar-6Car construct was 3D printed, resulting in an anthropomorphic, volumetric phantom (B) right after printing (day 0; scale in cm) and (C) on day 1, in relation to a one euro coin of 22.5 mm diameter. Whereas 3D printing of (D) the commonly used bioink 3Alg-9MC resulted in (E) a very good resolution (day 0; scale in cm) but also (F) a decrease in height of the construct after day 1, in relation to a one euro coin, the MR-active ink 3Alg-0.8Agar-6Car was capable of building up a stable brain phantom, demonstrated via *z*-height monitoring over the course of 100 days postfabrication storage in a humidified chamber (Figure S7). Resolution of fine gyri and sulci structure of the brain anatomy was lower for the 3Alg-0.8Agar-6Car hydrogel compared to the alginate-methylcellulose ink (B and C vs E and F).

In order to achieve larger dimensions toward real anatomical geometries, a novel printer based on a large-scale CNC system was established as a low-cost-solution. Manual replacement of used-up small volume printing cartridges (30 mL each) with filled-up cartridges allowed the fabrication of constructs of a volume >120 mL. In order to allow stable volumetric dimensions based on deposition of stable strands of reproducible shape, here, the blend with an intermediate level of 0.8% pre-cross-linked agarose (3Alg-0.8Agar-6Car) was chosen for fabrication of the brain phantom (Figure 12A). The print that took 6.5 h resulted in a large-scale phantom model with adequate shape fidelity, i.e., the overall brain shape along with some gyri morphology was identified (Figure 12B). However, throughout the printing process, several manual adjustments had to be made to ensure a homogeneous extrusion. This construct based on 3Alg-0.8Agar-6Car appeared geometrically stable on day 1 (Figure 12C) and over a long period of time (Figure S7, observed until day 100).

This MR-active ink was tested in direct comparison to a previously presented, specific bioink for clinically relevant dimensions, 3Alg-9MC, cross-linked by 0.1 M CaCl_2_. This ink allows high shape fidelity but the printing time (9.5 h) was even longer. Although it results in a high shape fidelity (Figure 12E), the construct showed a decreasing height already on day 1 (Figure 12F), due to the lower total polymer content or the reduced cross-linker concentration, chosen to prevent possibly cytotoxic concentrations for embedded cells (here: cell-free ink). The 3Alg-9MC blend, however, allowed a higher resolution and shape fidelity of details after printing and over time.

Here, like throughout the study, a nozzle diameter of 840 μm has been used. The rather long run times can be reduced by balancing speed and resolution for phantoms where resolutions below 1 mm are not necessarily required or available measurement devices would not be able to resolve such small structures. By using a 1200 μm diameter nozzle, the run times can be reduced by approximately 30–40%.

### 3.9 3D Plotting and *T*_1_ and *T*_2_ Measurements of a Large-Scale Biphasic Anthropomorphic Phantom

**Figure 13:**
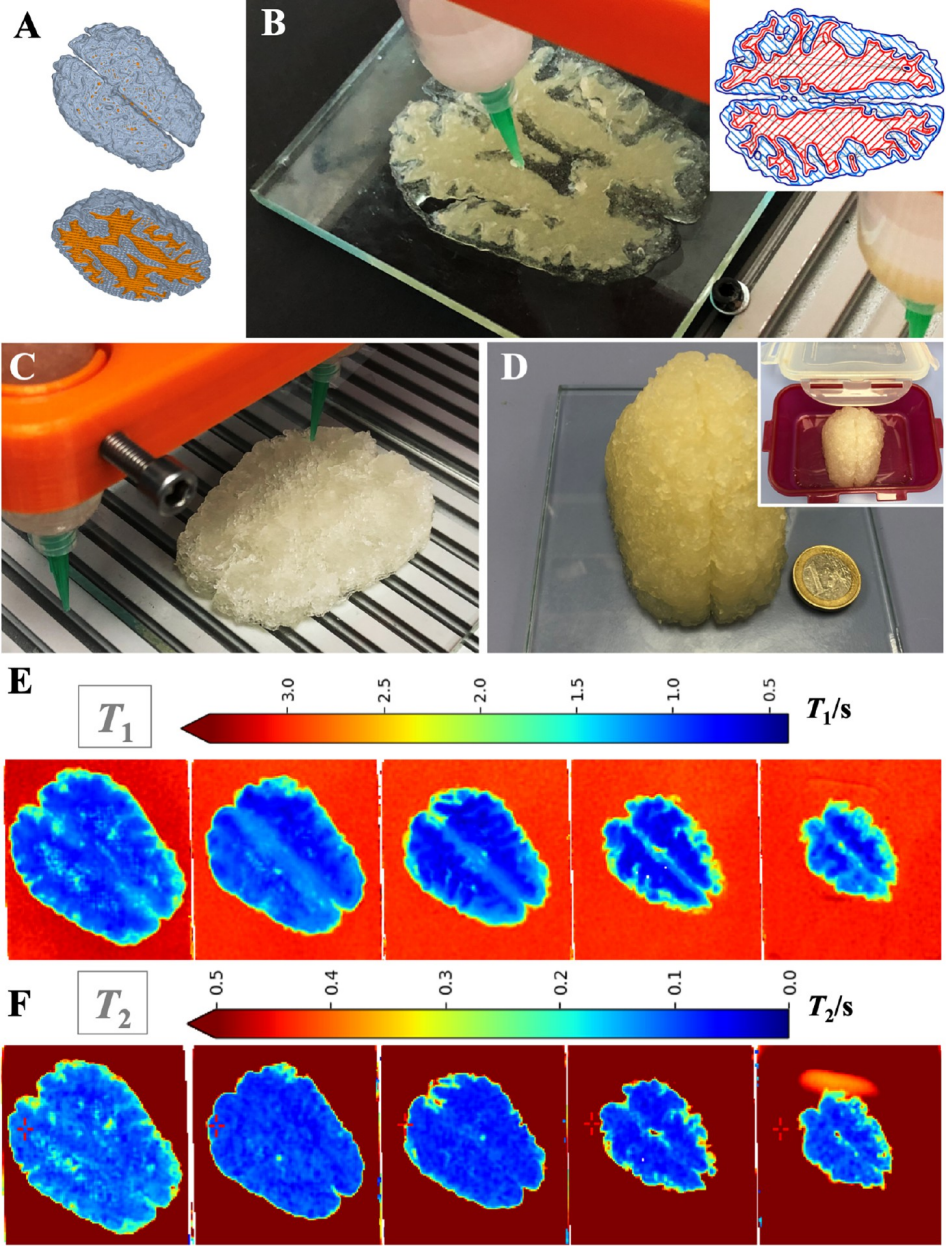
Large-scale printing and *T*_1_ and *T*_2_ determination in a biphasic anthropomorphic phantom (dimensions: approximately 100 mm × 60 mm × 44 mm, > 150 mL ink) mimicking GM (3Alg-0.5Agar-6Car), WM (3Alg-0.8Agar-6Car (280)), and CSF (approximately 350 mL of 10 mM CaCl_2_). (A) Schematic representation of the biphasic CAD model with GM and WM in gray and orange. (B) First five layers of a test print with one transparent and one opaque alginate-based ink, inset: printing pattern in one layer. (C, D) Fabrication, postprocessing, and container assembly (inset in D) of the final biphasic phantom, for which relaxation times were measured 2 days postfabrication. (E, F) Visual representation (color scales above, in s) of relaxation times of the biphasic (with CSF mimic: triphasic) 3D phantom from the bottom (left) to the top (right) for (E) *T*_1_ and (F) *T*_2_, both in s.

In the final experiment, the upscaling of anthropomorphic phantoms was combined with two-material printing on the customized Stepcraft printer, with two components based on 3Alg-0.8Agar-6Car (280) for WM and 3Alg-0.5Agar-6Car for GM and with NMR measurements afterward. In a sealable plastic container, a third component as CSF mimic consisting of a 10 mM CaCl_2_ solution was added (approximately 350 mL). Segmentation and modeling were successfully adjusted for two materials (Figure 13A), a model slice was printed using two materials with different opaque and transparent properties to allow visual distinguishing (Figure 13B), with an outline printed for each of the material phases (Figure 13B, inset). Later, the final biphasic phantom based on the selected tissue-mimicking materials was fabricated (Figure 13C) and prepared for shipment and measurements in a sealable container (Figure 13D). On day 2, *T*_1_ values for respective optical slices of the biphasic phantom were detected in the range of 0.5–1 s for the WM and 1.5–2 s for GM, while *T*_2_ values were measured in the range of 0–0.1 s for the WM and 0.2–0.3 s for GM.

The exact values expected for NMR measurements at 3.0 T were 1221 ms (*T*_1_) and 80 ms (*T*_2_) for GM, and 832 ms (*T*_1_) and 110 ms (*T*_2_) for WM, respectively.^50^ Here, a clear tendency in the biphasic phantom was realized, whereas the exact values were not achieved yet. In other casting approaches, the achieved values were closer to the native tissue properties compared to our study. Therefore, more adjustments will be necessary. Homogenization and possible reduction of polymer content play a crucial role to ensure printability. This might as well require, for the current material system, a more thorough balancing (and compromising) of the printability, stability, and realized relaxation times, i.e., native *T*_1_ and *T*_2_ values might be met by risking printability and stability. In this context, it could be worthwhile to investigate a blend without agarose addition (risking decreased stability postfabrication) or a blend which is primarily based on agarose and is, therefore, printable at increased temperatures and can be cross-linked by cooling of the material instead of by addition of a calcium salt solution.

### 3.10 Future Developments to Bridge Gaps between Hydrogel Printing Strategies and MRI Phantom Fabrication: An Outlook

With the aid of extrusion-based hydrogel printing,^34^ the fabrication of individualized soft matter objects or patient-specific tissue models has become possible aside from the conventional FDM printing of thermoplastic polymers.^52^ The fabrication of MRI phantoms has been an important topic since the very start but is yet to be extended by such techniques to be applied for suitable individual geometry generation. This offers a huge opportunity to transfer methods developed for a niche of cell printing to a wider set of applications. With respect to phantom design, in contrast to the monophasic and biphasic casting techniques, 3D printing of highly viscous inks forming volumetric hydrogel objects allows the combination of material-specific zones, free design of individual geometries, and the consideration of open cavities inside the 3D structures. The latter can be achieved by highly viscous inks allowing the printing of overhanging structures on its own or by implementation of sacrificial support structures based on dissolvable biopolymer inks such as plain methylcellulose.^53^ The strategies adopted from extrusion-based bioprinting were proven as beneficial here for the combination of different hydrogel phases in a spatially defined manner, whereas other groups before had only explored possibilities of phantom printing through silicones and other synthetic materials in casted, monophasic patterns.^25^ With our strategy, we ensured that the relaxation times of GM and WM can be adjusted with a clearly distinguishable contrast without the need for additional barriers, with a resolution and accuracy comparable to previous systems without anatomical shape.^16,54^ Achieving this without an additional barrier zone reduces the risk of signal loss, susceptibility artifacts or signal distortion. Typical brain MRI scans have a resolution of 1 mm, which can be also achieved with such a 3D printing method.

One may argue that the extensive run and fabrication times needed for 3D printing is a major drawback in comparison to the suggested casting techniques. However, fabrication of molds can also be quite time-consuming. Also, ideally, full automatization of the large phantom printing process with optimized printing parameters is expected to make long printing times less problematic. With the AM technique in use, each object can be produced with individual shapes most easily, only mass production of identical shapes will remain faster by casting methods. Depending on the composition chosen, the introduced centrifugation step prior to printing can help to reduce air bubbles but can result in a segregation and heterogeneity of the blend. Therefore, the inks should be always carefully prepared in advance; as an alternative option, a degassing step with mild vacuum can be used after cartridge filling.

In order to better understand material and gelation characteristics after printing and cross-linking, further viscoelastic and mechanical characterization will be valuable in the future. The differences regarding shape fidelity postfabrication comparing the MR-active ink to a bioink were described for 3Alg-9MC and different Alg-Agar-Car blends. Partly this might be also due to the different cross-linking degree through the CaCl_2_ concentrations of 100 mM and 1 M, respectively, and demonstrates the importance of a sufficient concentration of the cross-linking solution for alginate-based hydrogels.^55^ Further stabilization over time was facilitated by storage in low concentration (0.01–0.1 M) of CaCl_2_. If needed, additional post-cross-linking regimes can be beneficial to keep Alg-networks stable over time, as described earlier for stabilization of magnetically induced deformation of such alginate-based inks.^36,56^

As this study marks a first step toward bridging biofabrication concepts and phantom generation, alternative polymer blends and gelation schemes (such as thermo-responsive cross-linking) can be considered in future studies. Other polymer systems that are not cross-linked with the addition of ions could be beneficial for the consideration of physiological conductivity and permittivity values, as the sodium alginate-based system after calcium cross-linking already resulted in superphysiological conductivity ranges (data not shown). Here, the focus was on the biphasic geometrical features and adjusted relaxation times. Regarding the paramagnetic doping, in other MRI phantoms the use of MnCl_2_ and nickel compounds has been propagated.^57,58^ For such additives, again the printability would need to be investigated as these require a higher concentration and would most probably result in altered viscosity or even cross-linking density, as shown for alternative cross-linking ions strontium^59^ and barium in alginate gels.^60^ Furthermore, potential toxicity needs to be considered during ink preparation, cartridge and phantom handling, the printing process, etc. Adjusting other biofabrication methods such as digital light processing (DLP)^61^ might make the upscaling challenges easier and faster. With the application of those methods and materials and potential translation to the latest fabrication methods of DLP applied for bioprinting,^61,62^ the effect toward *T*_1_ and *T*_2_ adjustments would need to be examined. Later, such findings can be translated to other tissue types such as the human heart^63^ or even developed toward dynamic phantoms.^64^ In that regard, the findings can be combined with other latest advanced strategies of reusable, refillable 3D-printed models^65^ and nonprinted material and phantom development;^19,66^ both approaches and fields can still benefit from each other.

## 4 Conclusions

In this study, we presented, to the best of our knowledge for the first time, an approach of extrusion printing of real multiphasic, anthropomorphic phantom models with adjustable MR relaxation times *T*_1_ and *T*_2_, entirely based on highly viscous biopolymer inks. Altogether, we established a novel concept as a basis for a new standard workflow regarding design, 3D printing-based fabrication, and *T*_1_ and *T*_2_ characterization of hydrogel-based brain MRI phantoms of different compositions, volume scales, and geometries. This can be a promising and decisive step in the context of future developments in the field of phantom fabrication. We demonstrated the suitability of the proposed ink compositions based on rather easily and sustainably accessible natural polymers (sodium alginate, agarose, and carrageenan) for printing of complex phantom models; this was technically quite easily achievable with common printing/bioprinting equipment. We further highlighted the adjustability of this blend toward mimicking of *T*_1_ and *T*_2_ relaxation times of GM and WM of the human brain by tuning the ink compositions. Without the need for an additional barrier between the two highly concentrated biopolymer hydrogel phases, the spatially defined combination of different zones within one construct with distinguishable relaxation times was possible. More research will be required for the fine-tuning of these relaxation times by ink compositions, independent definition of *T*_1_ and *T*_2_, and for the adjustments of other characteristics such as dielectric properties (conductivity, permittivity). With respect to upscaling approaches, we demonstrated the capability of a customized low-cost system for fabrication of larger objects with the aid of a commercially available CNC machine tool kit in combination with a home-built extrusion head. The presented approaches with future improvements can then be translated to other tissues by considering geometry and relaxation times of, for example, the cardiovascular region or breast tissue.^9,10^ In conclusion, this initial approach of translating bioprinting techniques into phantom research demonstrates the versatility and the potency of extrusion-based printing of cross-linkable hydrogels for MRI phantom fabrication.

## Supporting Information Available

Supplementary data regarding rheology, swelling, and long-term storage (Figures S1–S7) (PDF)
