# Supplementary material for: 3D Extrusion Printing of Biphasic Anthropomorphic Brain Phantoms Mimicking MR Relaxation Times Based on Alginate-Agarose-Carrageenan Blends
Source: ACS Appl Mater Interfaces. 2022 Oct 21;14(43):48397–415. doi: 10.1021/acsami.2c12872 (PMC9634698; doi:10.1021/acsami.2c12872)
Supplement: Supplementary file 1 — am2c12872_si_001.pdf [file am2c12872_si_001.pdf]

SUPPORTING INFORMATION

3D Extrusion Printing of Biphasic Anthropomorphic Brain Phantoms Mimicking MR Relaxation Times Based on Alginate-Agarose-Carrageenan Blends

David Kilian[1], Wolfgang Kilian[2], Adriano Troia[3], Thanh-Duc Nguyen[1], Bernd Ittermann[2], Luca Zilberti[3] & Michael Gelinsky[1]*

[1] *Centre for Translational Bone, Joint and Soft Tissue Research, Faculty of Medicine Carl Gustav Carus, Technische Universität Dresden (TUD), Dresden, Germany*

[2] *Physikalisch-Technische Bundesanstalt (PTB), Braunschweig and Berlin, Germany*

[3] *Istituto Nazionale di Ricerca Metrologica (INRiM), Turin, Italy*

* corresponding author(s):
Prof. Dr. Michael Gelinsky
michael.gelinsky@tu-dresden.de
Fetscherstrasse 74, 01307 Dresden, Germany

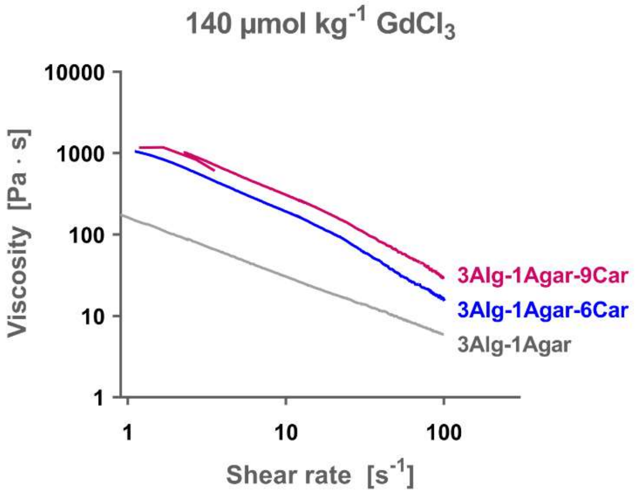

Figure S1: Shear-thinning behavior of the inks after $GdCl_3$ supplementation (140 µmol kg^{-1}) in an 3Alg-1Agar blend with different concentrations of carrageenan (0 %, 6 %, 9 %).

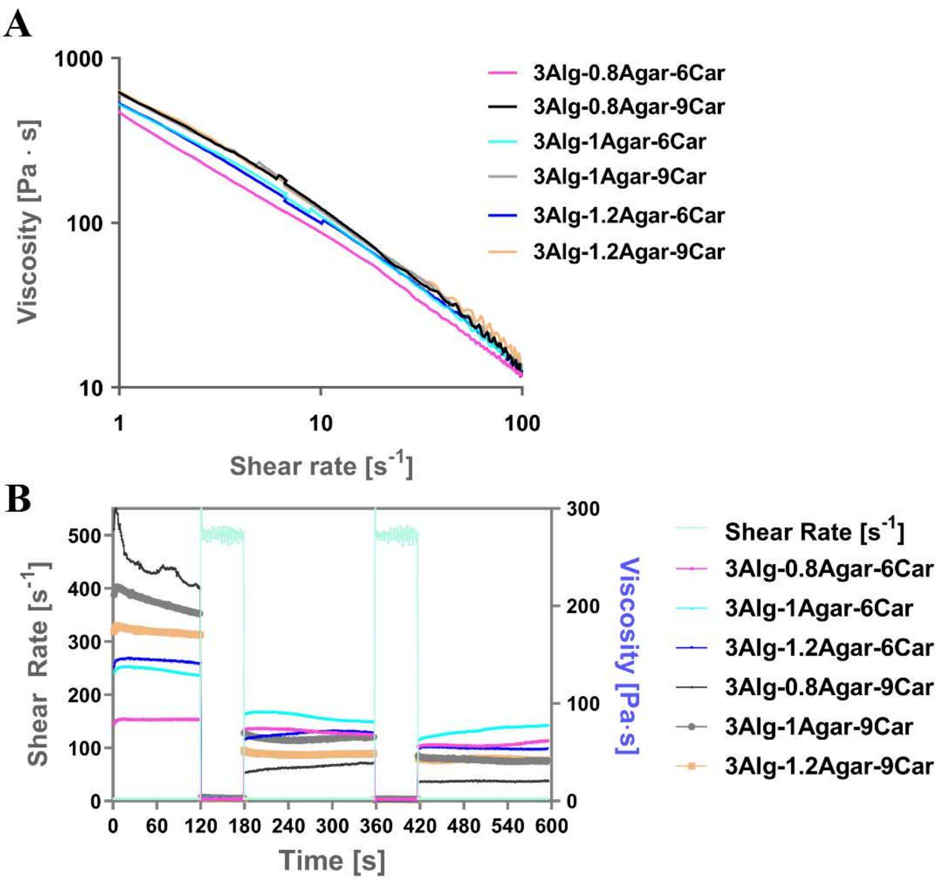

Figure S2: Rheological investigation of further ink compositions. Agarose and carrageenan content can be adjusted in a particular range by balancing the respective agarose-carrageenan concentrations. Representative curves of repeated measurements on a shear ramp (A) and shear recovery analysis (B) are shown.

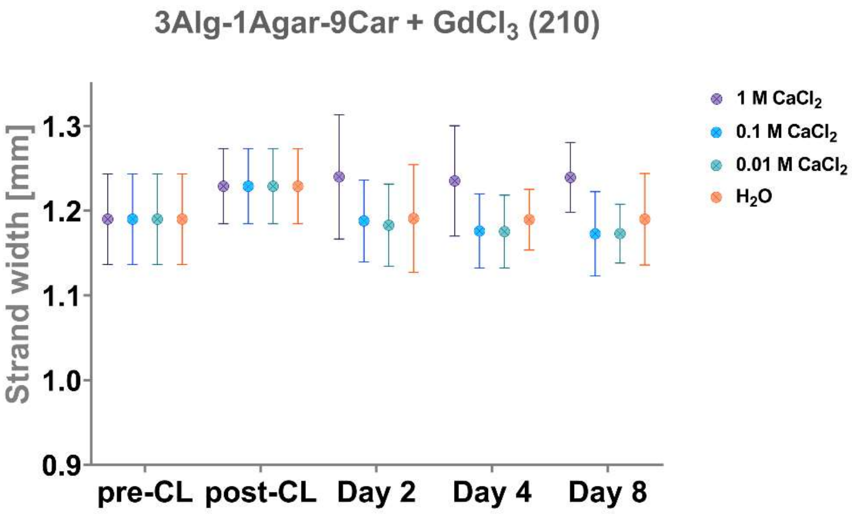

Figure S3: Swelling and shape fidelity in different storage solutions. The $GdCl_3$ (210 µmol kg^{-1})-supplemented ink 3Alg-1Agar-9Car (210) was used as an exemplary system to study the strand thickness in different storage conditions over a course of 8 days (mean ± SD, n ≥ 15). No significant changes in the investigated conditions were detected. Therefore, the storage solution can be chosen merely in accordance with ensuring the overall integrity of the constructs. For most applications, a $CaCl_2$ solution of 0.1 M was chosen, e.g. for the surrounding medium during T_1/T_2 measurements.

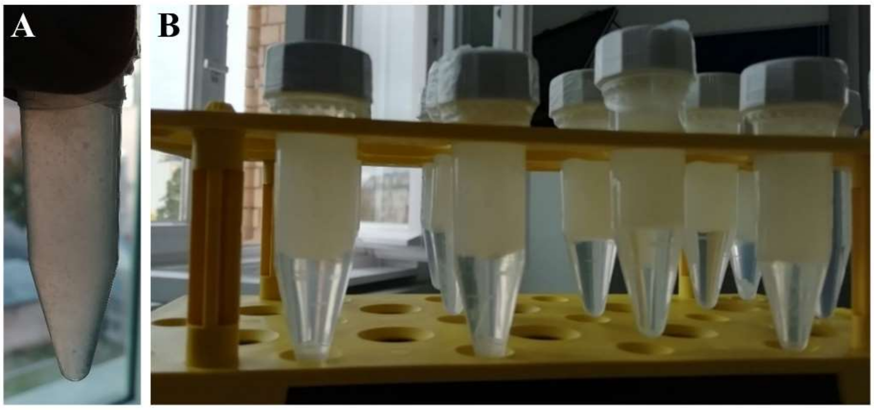

Figure S4: Eppendorf samples, stored and aligned for the T_1/T_2 measurements. (A) Example of an ink sample (non-printed, non-crosslinked). (B) 3D printed samples in tubes.

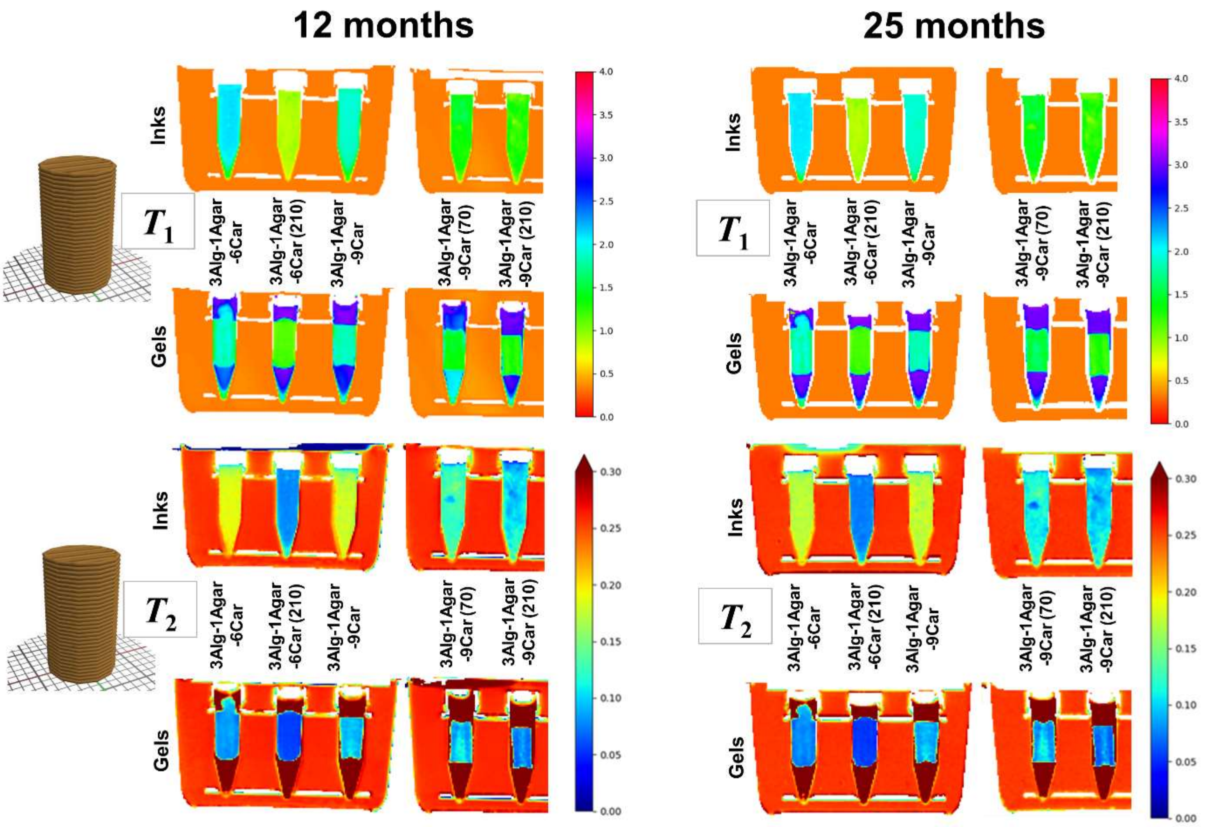

Figure S5: Visualization of relaxation times T_1/T_2 in mono-phasic Eppendorf samples, stored in Eppendorf tubes after 12 and 25 months (as graphed in Figure 6).

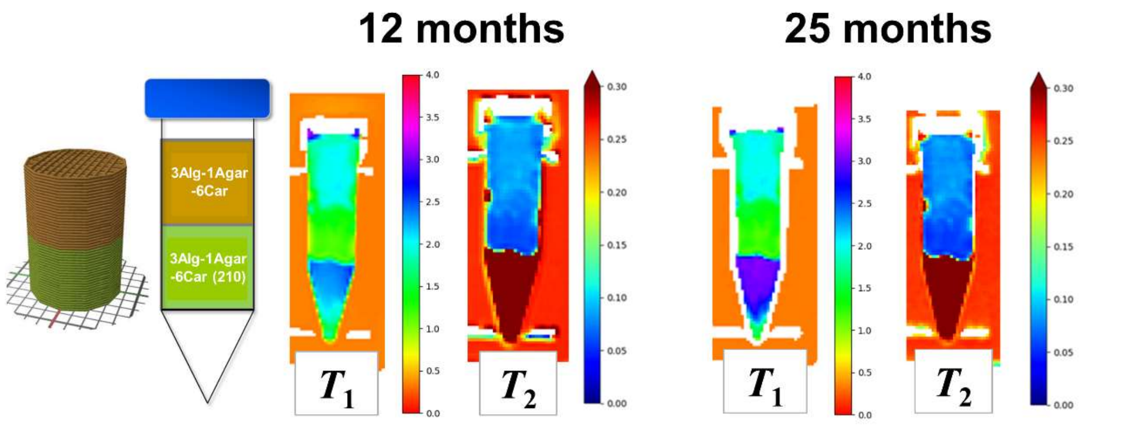

Figure S6: Visualization of relaxation times T_1/T_2 in biphasic samples, stored in Eppendorf tubes after 12 and 25 months (as graphed in Figure 7)

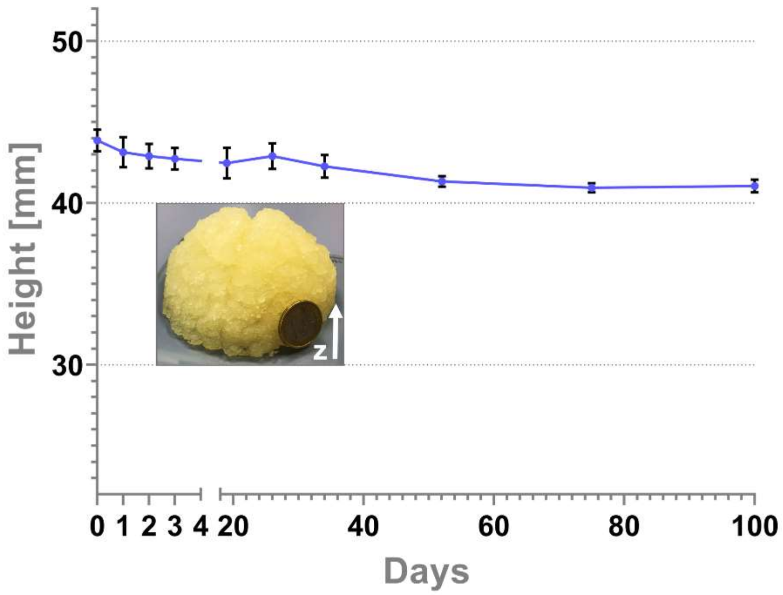

Figure S7: Maximum height of the printed 3Alg-0.8Agar-6Car brain phantom of large dimensions over the course of 100 days stored in a saturated humid atmosphere. The z-dimension remains stable after crosslinking with 1 M $CaCl_2$ solution (mean ± SD, n = 3; replicates of repeating measurements at different maximum height positions of one identical phantom). Inset image taken on day 1 after fabrication.
